# Supplementary material for: Why do people take part in atrial fibrillation screening? Qualitative interview study in English primary care
Source: BMJ Open. 2022 Mar 16;12(3):e051703. doi: 10.1136/bmjopen-2021-051703 (PMC8928318; doi:10.1136/bmjopen-2021-051703)
Supplement: Supplementary data [file bmjopen-2021-051703supp001.pdf]

SAFER study patient interview topic guide

Introduction and Consent Process

- Researcher to introduce themselves and check understanding of study and processes
- Informed consent and permission to audio record the interview obtained from the participant and partner (or other) if present
- (If applicable) - Explain to patient that this interview is like the previous interview they completed but will now focus on either their views about the screening invitation or their experience of screening.
- Select schedule based on time point of interview.

Interview 1 (post SAFER study invitation)

A. Experiences of screening

I'd like to start by thinking quite generally about screening programmes. There are national screening programmes for many different conditions – things like breast cancer, bowel cancer, and abdominal aortic aneurysm screening. What do you think about screening programmes?

Prompts:

- *Why do you think screening programmes are offered by the NHS?*
- *Potential benefits of screening*
- *Potential harms of screening*

Have you had experience of one of these screening programmes? I'm not asking for you to reveal personal information, just wanting to see if you had decided to go along to screening for something in response to an invitation.

Prompts:

- *Positive or negative experiences [if seemingly acceptable]*

B. Focus on atrial fibrillation

OK, we are going to move on to thinking about this particular condition – atrial fibrillation.

Had you ever heard of atrial fibrillation before you received the study invite? I'm not expecting you to know anything about it, just wanted to check in in case you do.

Prompts:

- *Does anyone they know have AF?*
- *Have they heard anything about AF from any other source?*

[Researcher to explain about atrial fibrillation]:

"Atrial fibrillation (AF) is a condition where the heart has an irregular beat. It may not cause symptoms, occurs in up to 10% of people over 65 years old. AF greatly increases risk of stroke by increasing the chance of blood clots developing. However, there is treatment that inhibits blood clotting (an anticoagulant, sometimes known as 'blood thinners'), and reduces the risk of stroke. About 10% of strokes occur in people unaware that they have AF."

What do you think having AF might mean for someone?
Prompts:
- *What it might be like to have an irregular heart beat*
- *Fears or concerns about living with AF*

What would you think about taking regular 'blood thinning' medication for AF?
Prompts:
- *An example medication is warfarin – what do you think about this medication?*

What do you think about the link AF has with stroke?
Prompts:
- *What experiences do people have with stroke?*
- *What are people's reactions to or concerns about stroke?*
- *What might it be like living with AF, knowing it increases the risk of stroke?*

C. Any other areas of discussion

Do you have any other areas, which we have not yet covered, which you would like to discuss in relation to this topic?

Interview close

- Thank participants for taking part in the study and remind them that their responses will remain confidential
- Ask participants if they have any questions.
- Let participants know they can contact the research team at any time using the details on the information leaflet, including if /d they have any further questions or wish to remove their responses from the study.

Interview 2 (post screening invitation)

A. Screening for atrial fibrillation

As you know, this interview is part of a research study we are undertaking to explore the offer of screening for AF in primary care. What were your first impressions on receiving the invitation to be screened for AF?

Prompts

- *Did you know anything about AF before you received the screening invite?*
- *Did you feel there was sufficient information in the screening invite?*

Do you think there might be any benefits of screening for AF?

Prompts:

- *Potential treatment of AF*
- *Stroke prevention*

Do you think there might be any harms or negative consequences of screening for AF?

Prompts:

- *Potential harms: psychological, false-positives, overdiagnosis, overtreatment, reduced quality of life, financial, opportunity costs*

Do you think this screening is relevant to you?

Prompt:

- *If so, why?*
- *If not, why not?*

Are you planning to go along for screening?

Prompt:

- *If so, why?*
- *If not, why not?*

Would you change anything about your life if screening showed you did have AF?

Prompt:

- *Lifestyle changes (routine/exercise/diet/smoking)*

B. Any other areas of discussion

Do you have any other areas, which we have not yet covered, which you would like to discuss in relation to this topic?

Interview 3 (Post screening)

A. Experiences of screening for AF

I'd like to explore with you your experiences of the AF screening programme. What were your first impressions on receiving the invitation to be screened for AF?

Prompts

- *Did you know anything about AF before you received the screening invite?*
- *Did you feel there was sufficient information in the screening invite?*
- *Did you feel the screening was relevant to you?*
- *If declined, why was this? What were your concerns about the process?*

Now I'd like to talk a little bit about what happened during the screening process [to be asked of screening attenders]

Prompts

- *What prompted you to go along?*
- *Did you understand what screening might involve?*
- *Did the doctor/nurse explain it all clearly to you, do you think?*
- *How did you find using the Zenicor device?*
- *Did you have any particular difficulties or challenges taking the readings you were asked to complete?*
- *Were you concerned about anything during the screening process? What was this in particular? Did you let anyone know about your concerns? Were these concerns addressed?*
- *What was good about your overall experience? Was there anything that was not so good about your experience?*
- *Did you understand how you were going to be informed about the outcome of your screening? If not, why not? How could things have been done differently?*
- *How did taking part in the screening make you feel?*

Given your experiences, do you think there are any benefits of screening for AF?

Prompts:

- *Potential treatment of AF*
- *Stroke prevention*

Do you think there might be any harms or negative consequences of screening for AF?

Prompts:

- *Potential harms: psychological, false-positives, overdiagnosis, overtreatment, reduced quality of life, financial, opportunity costs*

Would you recommend this screening programme to a find or family member?

Prompts:

- *If so, why?*
- *If not, why not?*

C. Any other areas of discussion

Do you have any other areas, which we have not yet covered, which you would like to discuss in relation to this topic?
