# Supplementary material for: Why do people take part in atrial fibrillation screening? Qualitative interview study in English primary care
Source: BMJ Open. 2022 Mar 16;12(3):e051703. doi: 10.1136/bmjopen-2021-051703 (PMC8928318; doi:10.1136/bmjopen-2021-051703)
Supplement: Author's manuscript [file bmjopen-2021-051703.draft_revisions.pdf]

BMJ Open

BMJ Open is committed to open peer review. As part of this commitment we make the peer review history of every article we publish publicly available.

When an article is published we post the peer reviewers' comments and the authors' responses online. We also post the versions of the paper that were used during peer review. These are the versions that the peer review comments apply to.

The versions of the paper that follow are the versions that were submitted during the peer review process. They are not the versions of record or the final published versions. They should not be cited or distributed as the published version of this manuscript.

BMJ Open is an open access journal and the full, final, typeset and author-corrected version of record of the manuscript is available on our site with no access controls, subscription charges or pay-per-view fees (http://bmjopen.bmj.com).

If you have any questions on BMJ Open's open peer review process please email info.bmjopen@bmj.com

BMJ Open

Why do people take part in atrial fibrillation screening? Qualitative interview study

Journal:	*BMJ Open*
Manuscript ID	bmjopen-2021-051703
Article Type:	Original research
Date Submitted by the Author:	01-Apr-2021
Complete List of Authors:	Hoare, Sarah; The Healthcare Improvement Studies Institute, Department of Public Health and Primary Care, University of Cambridge<br>Powell, Alison; The Healthcare Improvement Studies Institute, Department of Public Health and Primary Care, University of Cambridge<br>Modi, Rakesh; University of Cambridge Primary Care Unit, Department of Public Health and Primary Care, University of Cambridge<br>Armstrong, Natalie; University of Leicester, Department of Health Sciences<br>Griffin, Simon; University of Cambridge Primary Care Unit, Department of Public Health and Primary Care, University of Cambridge<br>Mant, Jonathan; University of Cambridge Primary Care Unit, Department of Public Health and Primary Care, University of Cambridge<br>Burt, Jenni; The Healthcare Improvement Studies Institute, Department of Public Health and Primary Care, University of Cambridge<br>SAFER Authorship Group, The
Keywords:	PRIMARY CARE, CARDIOLOGY, PUBLIC HEALTH, QUALITATIVE RESEARCH

SCHOLARONE™
Manuscripts

I, the Submitting Author has the right to grant and does grant on behalf of all authors of the Work (as defined in the below author licence), an exclusive licence and/or a non-exclusive licence for contributions from authors who are: i) UK Crown employees; ii) where BMJ has agreed a CC-BY licence shall apply, and/or iii) in accordance with the terms applicable for US Federal Government officers or employees acting as part of their official duties; on a worldwide, perpetual, irrevocable, royalty-free basis to BMJ Publishing Group Ltd ("BMJ") its licensees and where the relevant Journal is co-owned by BMJ to the co-owners of the Journal, to publish the Work in this journal and any other BMJ products and to exploit all rights, as set out in our licence.

The Submitting Author accepts and understands that any supply made under these terms is made by BMJ to the Submitting Author unless you are acting as an employee on behalf of your employer or a postgraduate student of an affiliated institution which is paying any applicable article publishing charge ("APC") for Open Access articles. Where the Submitting Author wishes to make the Work available on an Open Access basis (and intends to pay the relevant APC), the terms of reuse of such Open Access shall be governed by a Creative Commons licence – details of these licences and which Creative Commons licence will apply to this Work are set out in our licence referred to above.

Other than as permitted in any relevant BMJ Author's Self Archiving Policies, I confirm this Work has not been accepted for publication elsewhere, is not being considered for publication elsewhere and does not duplicate material already published. I confirm all authors consent to publication of this Work and authorise the granting of this licence.

For peer review only

Why do people take part in atrial fibrillation screening? Qualitative interview study

Sarah Hoare[1] (BA, MPhil, PhD. Research Associate. https://orcid.org/0000-0002-8933-217X)

Alison Powell[1] (MA, PhD. Research Associate. https://orcid.org/0000-0003-2524-5357)

Rakesh Modi[2] (MBBS MA MRCP MRCGP DCH DRCOG MSc. Academic GP and Wellcome Trust PhD Fellow. ORCID: https://orcid.org/0000-0001-9651-6690)

Natalie Armstrong[3] (BA, MSc, PhD. Professor of Healthcare Improvement Research. https://orcid.org/0000-0003-4046-0119)

Simon Griffin[2] (MBBS MSc DM FRCGP FMedSci Professor of General Practice. http://orcid.org/0000-0002-2157-4797)

Jonathan Mant[2] (MD, FFPH. Professor of Primary Care Research. https://orcid.org/0000-0002-9531-0268)

Jenni Burt[1] (PhD. Senior Social Scientist. https://orcid.org/0000-0002-0037-274X)

and The SAFER Authorship Group.

[1]THIS Institute (The Healthcare Improvement Studies Institute), University of Cambridge, Cambridge Biomedical Campus, Clifford Allbutt Building, Cambridge CB2 0AH, UK

[2]Primary Care Unit, Department of Public Health & Primary Care, University of Cambridge School of Clinical Medicine, Strangeways Research Laboratory, University of Cambridge, Worts Causeway, Cambridge CB1 8RN, UK

[3]SAPPHIRE Research Group, Department of Health Sciences, University of Leicester, George Davies Centre, University Road, Leicester LE1 7RH, UK

Correspondence to: Sarah Hoare, THIS Institute, University of Cambridge, Cambridge Biomedical Campus, Clifford Allbutt Building, Cambridge CB2 0AH, UK. Sarah.hoare@thisinstitute.cam.ac.uk

Word count: abstract: 298; manuscript: 3999

Number of references: 69

Number of figures: 1 **Number of tables:** 1

Statements

Acknowledgements

The SAFER Authorship Group includes:

Andrew Dymond, University of Cambridge, UK

Richard Hobbs, University of Oxford, UK

Rachel Johnson, University of Bristol, UK

Richard McManus, University of Oxford, UK

Kate Williams, University of Cambridge, UK

We thank the SAFER participants for their time and for sharing their experiences of taking part in screening. We also thank the GP practices involved and the local National Institute for Health Research Clinical Research Network for supporting this study. We are grateful to the SAFER study team and in particular to Millie Watson for her work in administering these interviews, and to James Brimicombe for managing the electronic SAFER data.

Ethical approval

The study was approved by the London-Central NHS Research Ethics Committee (reference number: 18/LO/2066).

For peer review only

Why do people take part in atrial fibrillation screening? Qualitative interview study

Abstract

Objectives: There is insufficient evidence to support national screening programmes for atrial fibrillation (AF). Nevertheless, some practitioners, policy-makers and special interest groups have encouraged introduction of opportunistic screening in primary care in order to reduce the incidence of stroke through earlier detection and treatment of AF. The attitudes of patients towards AF screening are unknown. We aimed to explore why people take part in AF screening.

Design: Semi-structured longitudinal interview study (n=53) of participant engagement in the SAFER study (Screening for Atrial Fibrillation with ECG to Reduce stroke). Interviews explored participants' experience of and attitudes towards AF screening. We undertook initial interviews face-to-face, with up to two follow-up interviews by telephone during the screening process. We analysed the interviews thematically.

Setting: 5 primary care practices in the East of England, UK.

Participants: 23 people taking part in the first feasibility phase of the SAFER study.

Results: Participants were supportive of screening for AF, describing it as a 'good thing to do'. Participants suggested screening could facilitate earlier diagnosis, more effective treatment and a better future outcome, despite most being unfamiliar with AF. Participating in AF screening helped attenuate participants' concerns about stroke, and demonstrated their commitment to self-care and being a 'good patient'. Participants felt that the screening test was not invasive and they were unlikely to have AF; they therefore considered engaging in AF screening was low risk, with few perceived harms.

Conclusions: Participants assessed the SAFER AF screening programme to be a legitimate, relevant and safe screening opportunity, and complied obediently with what they perceived to be a recommendation to take part. Their unreserved acceptance of screening benefit and lack of awareness of potential harms suggests that uptake would be high but reinforces the importance of ensuring participants receive balanced information about AF screening initiatives.

Keywords: screening, qualitative research, cardiovascular disease

For peer review only

Strengths and Limitations:

- Our research adds to the limited evidence base about atrial fibrillation (AF) screening participation: opportunistic AF screening is encouraged in primary care to reduce the incidence of stroke through earlier detection and treatment of AF, despite insufficient evidence currently available to support systematic AF screening.
- We report the views of people taking part in AF screening as part of a research study, limiting the relevance of our findings for understanding patient engagement in either opportunistic AF screening conducted as part of routine primary care or potential future systematic AF screening programmes.
- This was a study of people who participated in AF screening and it does not address the views of those who were invited and opted to not take part.
- The lack of ethnic diversity in our sample reduces the utility of the results, particularly because attitudes towards screening and AF are known to differ by ethnic background.
- Our research contributes to social science literature about patients' 'moral obligation' to participate in screening even when both the programme and the condition are largely unfamiliar to participants.

For peer review only

Introduction

Opinion on whether there should be a screening programme for Atrial Fibrillation (AF) is divided (1–3). Screening advocates include clinical societies (4–7), patient associations (8), clinicians (9,10), and pharmaceutical and technology companies (11,12). Justifications for AF screening are well-rehearsed. Having AF (a common, often asymptomatic, heart arrhythmia) increases the risk of stroke five-fold (13) with AF-related stroke typically more severe than non-AF-related stroke (14). AF anticoagulant treatment is effective at reducing stroke risk (15), and screening devices for use in the community are inexpensive (2). Conversely, caution around AF screening is attributed to insufficient evidence on effectiveness, cost-effectiveness and potential harms of national systematic AF screening programmes, which are not currently recommended (16,17). Questions remain, for example, about the difference in stroke risk and anticoagulation benefit for people with AF detected by screening rather than in routine care (16,18).

In the meantime, opportunistic AF screening is encouraged in primary care, leading to accusations of ‘back-door’ screening (12). Opportunistic screening occurs through many routes. In the UK, clinicians are recommended to assess patients’ pulse rhythm as part of NHS health checks (19), driven by an explicit aim of NHS programmes and policy to increase identification and diagnosis of previously undetected AF (20,21). NHS England has distributed digital ECG devices to increase AF detection (22), and some clinical commissioning groups have encouraged opportunistic AF case-finding in primary care (23,24).

Patient views have largely been absent from this debate. Patient associations which champion AF screening provide important information from the perspective of a motivated minority, but tell us little about the opinions of the wider public (12). If typically positive attitudes to screening programmes apply here (25,26), it seems likely that patients will be enthusiastic about AF screening, as seen in a small-scale study of AF screening trial participants (27) and informal reports from a previous AF screening trial (28). However, little is known about the reasons why patients are motivated to participate in AF screening (27,28). We aimed to explore why participants in SAFER, an AF screening study, opted to take part.

Methods

Design and participants

Our data are from a longitudinal interview study with participants in the AF screening study SAFER (Screening for Atrial Fibrillation using ECG to Reduce stroke) (https://www.safer.phpc.cam.ac.uk/). We interviewed participants during their study involvement to explore their experience of AF screening, including their views on why they had taken part.

The SAFER programme is ongoing, and interviews were conducted as part of a feasibility study. Participants were drawn from five participating GP practices in the East of England; eligible patients were aged 65 years and over, not taking anticoagulant medication, and were neither on a palliative care register nor living in a residential (care) home. Participants were first invited to contribute to SAFER research and subsequently to take part in AF screening. The screening involved using a Zenicor (www.zenicor.com) hand-held single-lead ECG device four times a day for between one and four weeks.

For peer review only

We selected interview participants from the first wave of feasibility phase participants. Potential interviewees were purposively sampled by age, gender and GP practice to ensure a varied sample. All participants were invited by letter and agreed to take part in SAFER and, on invitation to screening, elected to take part.

Data collection

We interviewed participants up to three times throughout their screening. Interview one occurred shortly after participants agreed to take part in the SAFER research study; interview two after a participant had received their invitation to take part in SAFER screening; and interview three after they had completed the screening. Interview one was held either at the participant's home or at their GP practice, and interviews two and three were conducted by telephone. Not all participants took part in all interviews: participants could elect not to take part in each interview phase, and some participants were not invited to the second interview to ensure that they had the opportunity to participate in screening (see Figure 1). Interviews were typically held with only the interviewer ([author 1]) and interview participant, but when participants' partners were present they were included in the discussion and consented to take part. Table 1 lists participants' characteristics (excluding partners).

Interviews were semi-structured and used a flexible topic guide exploring experiences of, and attitudes towards, screening in general and AF screening in particular. Consent was taken prior to each interview. Interviews were conducted in 2019, took approximately 40 minutes each and were audio-recorded.

Analysis

Interviews were transcribed verbatim and analysed using a thematic approach to explore the reasons why people took part in AF screening. The initial analysis phase focused on 16 transcripts selected for their relevance and drawn from all three interview time-points. [Author 1] conducted coding, on paper and then supported by the software NVivo 12, with codes generated inductively from topics raised by participants and deductively from the interview schedule. We developed key themes through consensus meetings between [authors 1, 2, 7]. The rest of the dataset was then reviewed by [author1] to establish the veracity of key themes and identify deviant cases, with the themes subsequently refined. These themes were synthesised to understand shared views of screening participation, aided by reference to social science and health screening literature.

[Figure 1: Flowchart of patient interviews]

[Table 1: Socio-demographics of participants]

Patient and public involvement (PPI)

The SAFER programme is guided by four PPI representatives who have been involved since the programme was conceived and funded. The representatives are embedded within the team (e.g. one is a co-applicant, another sits on the programme steering committee) and advise on all aspects of the design, management and delivery of the programme, including interpreting and disseminating the findings. We have also established a supplementary qualitative work-stream PPI group with whom we regularly consult about research design questions.

Results

We completed 53 interviews with 23 participants (interview 1 n=23, interview 2 n=10, interview 3 n=20). Engagement with AF screening within the SAFER study was driven by several inter-connected considerations, many relating to wider attitudes that screening is a

For peer review only

‘good thing’ to participate in, and to the perceived importance of preventive health behaviours. Participants knew little about AF, but were motivated to be screened by the perceived potential for reducing their risk of catastrophic disability associated with stroke, and a seemingly low-risk screening test with few if any apparent harms and significant perceived benefits.

The importance of screening

Participants were familiar with national screening programmes, often referring to their own or a relative’s prior experience of taking part in screening. Screening was described as a “no-brainer” by several participants, and it was clear within our sample that screening was seen as a routine healthcare activity in which participation was the only reasonable option. This option was driven by a desire to follow clinical recommendations, gain insight into health status and, if required, access early treatment, and was further underpinned by a ‘fear of missing out’ by not participating.

Clinically recommended

Screening invitations were typically seen as legitimate requests from a clinical authority which was presumed to recommend the screening programme. Participants explained that this meant that screening was something they ought to at least consider engaging with, and for a minority as a mandate that they must comply with.

“And I assume when something like that [a screening invitation] comes from the post, I immediately comply, I don’t question. I don't question because it's come from a...you know, it's a bit different to somebody trying to ring me up and telling me that they can spend my pension more successfully than I can. It comes from an authoritative source, and I don't think there's any question of me not believing what the prospects are.”
(05B_1)

Reassurance

Participants expected screening would provide them with reassurance about their health. This could be definitive, with a negative test result providing “peace of mind” that the screened for condition was “something else you can tick off the list of things to worry about” (06E_1). Reassurance could also be more anticipatory, whereby participation in screening was protective of regret if in the future one did indeed get diagnosed with the condition but had chosen not to be screened. Simply attending screening also seemed to confer reassurance about healthiness, whereby participants additionally acknowledged responsibility for maintaining their health and preventing ill-health:

“But, I think, it’s a brilliant idea and the thing is though, that things can happen in between, so you’ve still got to be responsible for your own health.”
(25A_1)

Early diagnosis, early treatment, better outcomes

Participants were clear that early identification of a screened condition would enable preventive action or prompt treatment to avert ill-health or reduce disease severity. Participants placed different emphases on the importance of these potentialities (from prevention to treatment) but were united by a presumption that earliness was advantageous. By comparison, ‘late’ identification or treatment was associated with perceived poorer outcomes for individuals and the healthcare system. Participants often drew on this binary conceptualisation of early versus late identification to explain why it was sensible to take part in screening:

“I’d rather find something out, if there is a screening thing that can indicate early symptoms that can be caught early and something can be done about it, I think I’d much rather have

For peer review only

that option than sort of find out later, you know, further down the road that, well it's too late now." (16D_1)

Identifying silent conditions: robust test

Underlying perceived benefits of early diagnosis was participants' awareness that screening could identify a condition before symptoms could be noticed or manifested, or for which symptoms were absent (a 'silent' condition):

"I get this thing for the bowel screening, which isn't as much fun. And despite that, I've gone in for it, because as a layman, I simply have no idea what's going on inside my body. The fact I feel fit, doesn't mean I am fit, you know, from those sort of complaints. [I: So…] So the answer is, I've said yes to that [bowel cancer screening] and I would have said yes to this [AF screening]."
(11A_2)

The ability of screening to identify silent conditions led to a view amongst some participants that screening tests could provide a particularly in-depth or robust review of health. For a few this extended the scope beyond the screened condition; here, the SAFER AF screening came to be viewed as a "free medical" (15A_3). Screening thus offered ready access to healthcare and perceived "health checks" without the need to present tangible health symptoms:

R: "I think, I don't want to bother the doctor he's so busy, so I won't bother to go until I really think, I'm going to have to go. […] I think, a lot of people don't go, so screening, perhaps, would ensure that they are seen for a particular thing, once every three years or however they're done."
I: "And, do you think going to a screening appointment is different to just going for something else?"
R: "Well, I think, it has to be 'cause I don't think anybody, unless they've got any symptoms, has any reason to think they've got anything."
(25A_1)

Not taking part

Associated with reducing the risk of 'late' diagnosis, participating in screening also protected participants from moral judgement about *not* engaging in screening. Non-screeners were variously portrayed by participants as unduly embarrassed, uninformed, indolent, irresponsible, wilfully ignorant, or gratuitously anxious, and often a combination of these. A significant part of participants' moralising about non-screeners was concern that they were missing out on the benefit of screening:

"I mean maybe there are some people that don't want to know. They live in a dream world and…but I just think if you've got the chance to do something about it and it gives you a better quality, even if it's only a short one, that's great, you should grab it."
(15A_3)

AF screening

AF

AF was a new condition to most participants, except those with first-hand experience as a result of a family member's diagnosis or prior clinical knowledge. Some participants struggled to pronounce the term and many explained that they had conducted internet research to better understand the condition, supplementing the study literature provided. Despite this, after interviewer explanation, participants engaged with the concept of an irregular heartbeat and described what they thought it might be like to have the condition. At best, participants understood AF as a benign condition, but most expressed concern about

For peer review only

the association with stroke. Participants anticipated that, if they had AF, they would worry about having a major and sudden heart-related incident, and expected having to modify their lifestyle (for some to a very significant and limiting degree) and to receive corrective treatment to address it:

I: [...] "I wonder what you thought it might be like to have an irregular heartbeat?"

R: "A little bit disconcerting. I would say you would want it regulated, maybe if it's to have a pacemaker fitted or something. You wouldn't want to live with something that you know...you'd probably be able to feel it beating faster. You wouldn't want to think to yourself, am I having a stroke shortly? So I would say it's more peace of mind to know that you can regulate it if you have a... I mean, if you know that your heart is beating faster and it could be a start of a stroke, and you can take a pill to stop it and you didn't know about it, you'd be a bit miffed to say if you didn't have one."
(06E_1)

Screening
Most participants did not expect to receive a diagnosis of AF as a result of taking up the offer of screening. They explained this in relation to their own perception of their health, whether because they were healthy overall, or because they identified an absence of AF symptoms or prior heart-related issues. Despite this, participants explained that participating in screening was useful because there was a *chance* that they may unknowingly have AF. As a silent condition with perceived serious consequences and a presumption for curative treatment, participants recognised the utility of checking to see if they had it:

I: "Do you think the atrial fibrillation screening is relevant to you?"
R: "I don't suffer from any of those symptoms. That's as far as I can...or I'm not aware of suffering from those symptoms. That's as far as I can go. Like a lot of things that are more evident with age, I think it ill behoves me to say, oh, I'm, you know, I don't need to be tested because I feel fine."
(11A_2)

Stroke fear
Participants were clear that stroke was not a condition they wished to experience. The consequences of stroke were recognised to be potentially debilitating and life-changing. Whilst those who knew people who had had a stroke often recognised a diversity of recovery experiences that included more positive outcomes, there remained a concern about the high level of disability stroke could cause.

Participants typically recognised both that a stroke could happen unexpectedly "to anybody at any time, any day, any age really" (16D_1) and that there were genetic and modifiable lifestyle factors which could increase the risk of having a stroke. Together with concern about the consequences of having a stroke, this dual conception of stroke as preventable and unexpected made the association between AF and stroke significant, and thus AF screening particularly worthwhile:

I: "What do you think about the link between atrial fibrillation and stroke?"
R: "I would try to do everything I can not to have a stroke. So if there was something I could do that would stop me turning into a vegetable, I will do it. So as far as I'm concerned, it's a no-brainer. Once you've had the stroke, whatever life you have got left, it's not going to be much fun, so try and avoid it."
(06E_1)

Low-risk

For peer review only

The AF screening test involved participants placing their thumbs or two fingers on a portable ECG device. Participants unanimously recognised this to be non-invasive and typically understood the screening to be safe and to not cause harm, often aligning it with the ease of measuring blood pressure.

Participants acknowledged that anxiety around the screening test may arise for others. However, they frequently related this to the screened individuals' psychological state rather than something inherent to the testing process. Participants rarely recognised other iatrogenic screening harms. If they did, such harms were presented as legitimate concerns and often as intrinsic to testing. However, participants presumed the screening was safe and, when discussed, positioned consideration of these issues as the responsibility of the screening provider.

Compared to the perceived fuzzy and inconsequential harms of screening, participants saw the advantages of AF screening as tangible and significant. Screening could identify AF 'early', using a test that was perceived to cause no harm, and which could ameliorate the risk of stroke:

R1: "Any harm? No I don't think that any harm can arise from that all, personally, screening, no."
R2: "I mean to say, it looks as if just by putting your thumbs on the actual screening test on the box, you're not going to get anything invasive from that. And, I say, if it shows up something that can be dealt with sooner rather than later, that can only be a good thing in our view really."
(16D_2)

Discussion

Summary

We explored the reasons why people took part in AF screening through interviews with SAFER study participants. Their accounts presented screening as a routine obligation and something one 'ought' to do as a responsible patient to ensure good health and prevent illness. Limited awareness of AF did not detract from their view of the utility of AF screening – rather, preventing stroke was a strong rationale for participating. Participants assessed AF screening to be a legitimate, relevant and safe screening opportunity, and complied obediently with what they perceived to be a recommendation to take part.

Strengths and limitations

Our research adds to the limited evidence about AF screening participation. Our findings also contribute to social science literature about patients' 'moral obligation' (29) to participate in screening (29–32), even when both the programme and the condition are largely unfamiliar to participants.

It is likely that our results reflect the delivery of SAFER as a research study. Whilst the SAFER study has been designed to mimic a national screening programme, participants were aware that it was research. This may limit the relevance of our findings for understanding patient engagement in either opportunistic AF screening conducted as part of routine primary care, or potential future systematic AF screening programmes. For example, participants reported that a key reason for taking part was to 'help out', a prevalent and well-recognised motivation for participating in research (33).

This was a study of people who participated in AF screening, and does not address the views of those who were invited and opted to not take part. Nevertheless, focusing on

For peer review only

people who did engage has allowed us to understand the information needs and potential misconceptions of this population.

Participants' familiarity with, and participation in, national screening programmes suggests their enthusiasm for AF screening will not be relevant to all screening invitees. For example, recent studies in the UK found that prior attendance in screening is positively correlated with future participation.(34,35) Participants in our study match national trends by being relatively affluent by practice deprivation status (36). The lack of ethnic diversity in our sample will also necessarily reduce the utility of the results, particularly because screening uptake and attitudes towards AF differ by ethnic background (36,37).

Comparison with existing literature

Participants' concern about having a stroke mediated their unfamiliarity with AF and encouraged them to take part. The novelty of AF is unsurprising as studies find limited awareness of the condition, whether the participants' relationship to AF is: just at risk (38); being screened for it (27); prior to diagnosis (39); or having it (37,40). Similarly, those with AF report being worried or fearful about their condition (39,40), consider it a serious heart disease (41) and overestimate the risk of stroke (42). AF was explained to participants in the context of stroke-risk, and this may explain participants' worries about AF-induced stroke: the majority of AF patients in a 2002 study (37) did not consider their condition to be severe, but almost half were not aware that having AF predisposed them to stroke.

The enthusiasm participants had for AF screening, and the weighting they attributed to the benefits versus the harms of screening, accord with other studies on patient experience of national screening programmes (43,44). There is widespread public support for screening even in survey scenarios in which there was no treatment for the screened condition (25,26), or if screening is not clinically recommended because participants are outside screening age thresholds (45,46). The benefits SAFER participants attributed to AF screening concur with reported experience. Screening offers the hope of reassurance (29,47,48) and confirmation of healthiness (47), or in the worst case, the benefit in knowing one's ill-health status (49) and associated advantageous early diagnosis or treatment (50). Participation also offers an 'insurance policy' (51), whereby the often presumed 'health check' (48) means patients can be assured that 'they had done everything they could' (47) if they later develop the screened condition. Like SAFER participants, this enthusiasm is contextualised by reported limited concern or awareness of screening harms (50). A recent US study found that many respondents could not name any harms of screening, and where they could, focused, as SAFER participants did (27), on the direct harms of the screening test itself (52). Even when harms are recognised, these are typically of a lower priority to participants compared to the benefits of screening (53).

Informing participants' consideration of the harms and benefits of screening was an assumption that taking part in screening was something they ought to do, corresponding with evidence from screening decision-making studies (46,54–57). Sociological work has shown how screening participation is associated with maintaining, and being responsible for, one's own health (29–32,47,48,58–60), and part of patients' efforts to be a 'good patient' and to use healthcare resources appropriately (61).

Implications for practice and research

Patients can be expected to be unfamiliar with AF, to anticipate that screening is recommended, to perceive any AF test as a comprehensive heart function review and to be positive about screening. Clinicians should be prepared to work with patients to help them understand what they are being offered and what the risks and benefits of taking part are. SAFER participants suggested that early identification leads to better results. Counselling patients on the benefits and risks of AF screening is particularly pertinent given participant

For peer review only

expectations for curative treatment and because clinical evidence on the benefit of managing screen-detected AF is still uncertain (18).

Receiving a positive result may be unexpected for those engaging in screening for health confirmation, and could engender feelings of health vulnerability (62). These participants may need help to moderate concerns about the severity and lifestyle impact of the condition. Clinician engagement is significant because their reactions and approaches to AF have been found to impact patients' perceptions of their condition (63), and patients' understanding of AF may be aided by often called-for patient education interventions (37,42,64,65). Negative test results may confer 'healthiness' (66) and induce false reassurance about their stroke risk. Though the evidence-base is small (67) it is plausible that patients' existing 'unhealthy' behaviours are validated as acceptable and continued (68), whilst any future AF-related symptoms may be 'downplayed' (69), discouraging prompt healthcare seeking. Consequently, clinicians are recommended to contextualise negative results with reference to the necessary limitations of any screening test and patients' ongoing risk of developing the condition (66).

Our study has contributed to a small evidence-base on patient experience of AF screening (27). If AF screening is demonstrated to be effective, then complementary studies would be important to understand the attitudes of people who do not participate in screening and how participants (particularly those with positive results) weigh up the benefits and harms throughout the screening process.

For peer review only

Tables

Table 1: Socio-demographics of participants (n=23)

Participant characteristic	*n*
**Age group**	
65-69 years	9
70-74 years	7
75-79 years	5
80+ years	2
**Gender**	
Female	10
Male	13
**Practice** (*Deprivation score 1-10; 10 = least deprived)*	
A *(10)*	8
B *(8)*	5
C *(8)*	4
D *(6)*	4
E *(5)*	2

For peer review only

Contributions

SH and JB drafted the original manuscript. All authors critically reviewed, revised and approved the final version. All members of the SAFER Authorship Group reviewed and approved the initial submission and had the opportunity to comment, and the final version was shared with them.

Competing interests

JM has received hororaria from BMS/Pfizer.

Funding

The SAFER study is funded by the National Institute for Health Research (NIHR) Programme Grants for Applied Research (grant reference number RP-PG-0217-20007) and School for Primary Care Research (SPCR-2014-10043, project 410). The views expressed are those of the authors and not necessarily those of the NIHR or the Department of Health and Social Care.

RM is supported by the Wellcome Trust as part of the Wellcome Trust PhD Programme for Primary Care Clinicians (grant reference number 203921/Z/16/Z).

NA is supported by a Health Foundation Improvement Science Fellowship (N/A award/grant number) and also by the National Institute for Health Research (NIHR) Applied Research Collaboration East Midlands (ARC EM) (N/A award/grant number). The views expressed are those of the authors and not necessarily those of the NHS, the NIHR or the Department of Health and Social Care.

The University of Cambridge has received salary support in respect of SG from the NHS in the East of England through the Clinical Academic Reserve (N/A award/grant number).

JM is an NIHR Senior Investigator (N/A award/grant number).

SH, AP and JB are based in The Healthcare Improvement Studies Institute (THIS Institute), University of Cambridge. THIS Institute is supported by the Health Foundation, an independent charity committed to bringing about better health and healthcare for people in the UK (N/A award/grant number).

Data sharing

The data may be available on request from the authors, subject to appropriate permissions: please email the PI Jenni Burt (jenni.burt@thisinstitute.cam.ac.uk) for details.

For peer review only

References

1. Neubeck L, Orchard J, Lowres N, Freedman SB. To Screen or Not to Screen? Examining the Arguments Against Screening for Atrial Fibrillation. Heart Lung Circ. 2017 Sep;26(9):880–6.

2. Lown M, Garrard J, Irving G, Edwards D, Hobbs FR, Mant J. Should we screen for atrial fibrillation? British Journal of General Practice. 2017 Jul;67(660):296–7.

3. Lown M, Moran P. Should we screen for atrial fibrillation? BMJ. 2019 Feb 13;l43.

4. Hobbs FR, Taylor CJ, Jan Geersing G, Rutten FH, Brouwer JR. European Primary Care Cardiovascular Society (EPCCS) consensus guidance on stroke prevention in atrial fibrillation (SPAF) in primary care. Eur J Prev Cardiol. 2016 Mar;23(5):460–73.

5. Kirchhof P, Benussi S, Kotecha D, Ahlsson A, Atar D, Casadei B, et al. 2016 ESC Guidelines for the management of atrial fibrillation developed in collaboration with EACTS. Eur Heart J. 2016 Oct 7;37(38):2893–962.

6. Lip GY, Ramsay SG. Insights from the RCPE UK Consensus Conference on approaching the comprehensive management of atrial fibrillation. Expert Review of Cardiovascular Therapy. 2012 Jun 1;10(6):697–700.

7. Authors/Task Force Members, Camm AJ, Lip GYH, De Caterina R, Savelieva I, Atar D, et al. 2012 focused update of the ESC Guidelines for the management of atrial fibrillationAn update of the 2010 ESC Guidelines for the management of atrial fibrillation Developed with the special contribution of the European Heart Rhythm Association. Europace. 2012 Aug 24;14(10):1385–413.

8. Atrial Fibrillation Association, AntiCoagulation Europe. The AF Report. Atrial Fibrillation: Preventing A Stroke Crisis [Internet]. Atrial Fibrillation Association and AntiCoagulation Europe; 2011. Available from: http://atrialfibrillationassociation.org.uk/app/webroot/files/file/140508-cw-FINAL-The%20AF%20Report.pdf

9. Robson J, Schilling R. Atrial fibrillation: time for active case finding. British Journal of General Practice. 2019 Feb;69(679):58–9.

10. Kearney M, Fay M, Fitzmaurice DA. Stroke prevention in atrial fibrillation: we can do better. Br J Gen Pract. 2016 Feb 1;66(643):62–3.

11. Mahase E. Atrial fibrillation groups lobby patients to overturn screening committee decision. BMJ [Internet]. 2019 Jul 9 [cited 2020 Oct 2];366. Available from: https://www.bmj.com/content/366/bmj.l4612

12. McCartney M, Fell G, Finnikin S, Hunt H, McHugh M, Gray M. Why ‘case finding’ is bad science. J R Soc Med. 2019 Dec 12;014107681989142.

13. Wolf PA, Abbott RD, Kannel WB. Atrial fibrillation as an independent risk factor for stroke: the Framingham Study. Stroke. 1991 Aug;22:983–8.

14. Marini C, De Santis F, Sacco S, Russo T, Olivieri L, Totaro R, et al. Contribution of atrial fibrillation to incidence and outcome of ischemic stroke: results from a population-based study. Stroke. 2005 Jun;36:1115–9.

For peer review only

15. G. Hart R, A. Pearce L, I. Aguilar M. Meta-analysis: Antithrombotic Therapy to Prevent Stroke in Patients Who Have Nonvalvular Atrial Fibrillation. Annals of Internal Medicine [Internet]. 2007 Jun 19 [cited 2020 Nov 20]; Available from: https://www-acpjournals-org.ezp.lib.cam.ac.uk/doi/abs/10.7326/0003-4819-146-12-200706190-00007

16. King S, Fitzgerald A, Bartlett C, Mahon J, Arber M, Carr E, et al. Evidence Summary for Screening for Atrial Fibrillation in Adults: External review against programme appraisal criteria for the UK National Screening Committee. London: UK National Screening Committee; 2019.

17. US Preventive Services Task Force, Curry SJ, Krist AH, Owens DK, Barry MJ, Caughey AB, et al. Screening for Atrial Fibrillation With Electrocardiography: US Preventive Services Task Force Recommendation Statement. JAMA. 2018 Aug 7;320(5):478.

18. Jones NR, Taylor CJ, Hobbs FDR, Bowman L, Casadei B. Screening for atrial fibrillation: a call for evidence. Eur Heart J. 2020 Mar 7;41(10):1075–85.

19. NHS Health Check: Best practice guidance [Internet]. London: Public Health England; 2017 [cited 2020 Oct 2]. Available from: https://www.england.nhs.uk/london/wp-content/uploads/sites/8/2019/08/2.1.7-NHS-Health-Check-Best-Practice-Guidance-2017.pdf

20. NHS RightCare » High value intervention in atrial fibrillation [Internet]. [cited 2020 Oct 9]. Available from: https://www.england.nhs.uk/rightcare/products/pathways/cvd-pathway/af/

21. NHS. The NHS Long Term Plan [Internet]. 2019 [cited 2020 Sep 23]. Available from: https://www.longtermplan.nhs.uk/wp-content/uploads/2019/08/nhs-long-term-plan-version-1.2.pdf

22. Independent Evaluation of the AHSN Network mobile ECG rollout programme: Full Report. Southampton: Wessex Academic Health Science Network; 2019 p. 82.

23. Cole J, Torabi P, Dostal I, Homer K, Robson J. Opportunistic pulse checks in primary care to improve recognition of atrial fibrillation: a retrospective analysis of electronic patient records. British Journal of General Practice. 2018 Jun;68(671):e388–93.

24. Smith T, Fell C, Otete H, Chauhan U. GP incentives to design hypertension and atrial fibrillation local quality-improvement schemes: a controlled before–after study in UK primary care. Br J Gen Pract. 2019 Oct 1;69(687):e689–96.

25. Schwartz LM, Woloshin S, Floyd J. Fowler J, Welch HG. Enthusiasm for Cancer Screening in the United States. JAMA. 2004 Jan 7;291(1):71–8.

26. Waller J, Osborne K, Wardle J. Enthusiasm for cancer screening in Great Britain: a general population survey. Br J Cancer. 2015 Feb 3;112(3):562–6.

27. Lown M, Wilcox CR, Hughes S, Santer M, Lewith G, Moore M, et al. Patients' views about screening for atrial fibrillation (AF): a qualitative study in primary care. BMJ Open. 2020 Mar 1;10(3):e033061.

For peer review only

28. Bury G, Swan D, Cullen W, Keane D, Tobin H, Egan M, et al. Screening for atrial fibrillation in general practice: A national, cross-sectional study of an innovative technology. International Journal of Cardiology. 2015 Jan 15;178:247–52.

29. Howson A. Cervical screening, compliance and moral obligation. Sociology of Health & Illness. 1999;21(4):401–25.

30. Bush J. “It’s just part of being a woman”: cervical screening, the body and femininity. Social Science & Medicine. 2000 Feb 1;50(3):429–44.

31. Ward PR, Coffey C, Meyer S. Trust, choice and obligation: a qualitative study of enablers of colorectal cancer screening in South Australia. Sociology of Health & Illness. 2015;37(7):988–1006.

32. Howson A. Embodied obligation: The female body and health surveillance. In: Nettleton S, Watson J, editors. The body in everyday life. ProQuest Ebook Central, http://ebookcentral.proquest.com: Taylor and Francis; 1998.

33. Sheridan R, Martin-Kerry J, Hudson J, Parker A, Bower P, Knapp P. Why do patients take part in research? An overview of systematic reviews of psychosocial barriers and facilitators. Trials [Internet]. 2020 Mar 12 [cited 2020 Oct 21];21. Available from: https://www.ncbi.nlm.nih.gov/pmc/articles/PMC7069042/

34. Rebolj M, Parmar D, Maroni R, Blyuss O, Duffy SW. Concurrent participation in screening for cervical, breast, and bowel cancer in England. J Med Screen. 2020 Mar;27(1):9–17.

35. McCowan C, McSkimming P, Papworth R, Kotzur M, McConnachie A, Macdonald S, et al. Comparing uptake across breast, cervical and bowel screening at an individual level: a retrospective cohort study. British Journal of Cancer. 2019;121(8):710–4.

36. Public Health England. Supporting the health system to reduce inequalities in screening. https://assets.publishing.service.gov.uk/government/uploads/system/uploads/attachment_data/file/816245/PHE_Screening_inequalities_strategy_2018__1_.pdf: Public Health England; 2019 p. 27. Report No.: GW-489.

37. Lip GYH, Kamath S, Jafri M, Mohammed A, Bareford D. Ethnic Differences in Patient Perceptions of Atrial Fibrillation and Anticoagulation Therapy. Stroke. 2002 Jan 1;33(1):238–42.

38. McCabe PJ, Barton DL, DeVon HA. Older Adults at Risk for Atrial Fibrillation Lack Knowledge and Confidence to Seek Treatment for Signs and Symptoms. SAGE Open Nurs [Internet]. 2017 [cited 2020 Oct 6];3. Available from: https://www.ncbi.nlm.nih.gov/pmc/articles/PMC6326385/

39. McCabe PJ, Rhudy LM, DeVon HA. Patients’ experiences from symptom onset to initial treatment for atrial fibrillation. Journal of Clinical Nursing. 2015;24(5–6):786–96.

40. Aliot E, Breithardt G, Brugada J, Camm J, Lip GYH, Vardas PE, et al. An international survey of physician and patient understanding, perception, and attitudes to atrial fibrillation and its contribution to cardiovascular disease morbidity and mortality. Europace. 2010 May;12(5):626–33.

For peer review only

41. Koponen L, Rekola L, Ruotsalainen T, Lehto M, Leino-Kilpi H, Voipio-Pulkki L-M. Patient knowledge of atrial fibrillation: 3-month follow-up after an emergency room visit. Journal of Advanced Nursing. 2008;61(1):51–61.

42. Mehawej J, Saczynski J, Gurwitz JH, Abu HO, Bamgbade BA, WANG W-J, et al. Self-reported risk of stroke and factors associated with underestimation of stroke risk among older adults with atrial fibrillation: the SAGE-AF study. J Geriatr Cardiol. 2020 Aug;17(8):502–9.

43. Hoffmann TC, Del Mar C. Patients' expectations of the benefits and harms of treatments, screening, and tests: a systematic review. JAMA Intern Med. 2015 Feb;175(2):274–86.

44. Gigerenzer G, Mata J, Frank R. Public knowledge of benefits of breast and prostate cancer screening in Europe. Journal of the National Cancer Institute. 2009 Sep 2;101(17):1216–20.

45. Kotwal AA, Walter LC, Lee SJ, Dale W. Are We Choosing Wisely? Older Adults' Cancer Screening Intentions and Recalled Discussions with Physicians About Stopping. Journal of General Internal Medicine. 2019;34(8):1538–45.

46. Torke AM, Schwartz PH, Holtz LR, Montz K, Sachs GA. Older adults and forgoing cancer screening: 'I think it would be strange'. JAMA internal medicine. 2013 Apr 8;173(7):526–31.

47. Willis K. Personal choice/social responsibility: Women aged 40–49 years and mammography screening. Journal of Sociology. 2004 Jun;40(2):121–36.

48. Willis K. "I Come Because I Am Called": Recruitment and Participation in Mammography Screening in Uppsala, Sweden. Health Care for Women International. 2008 Feb 11;29(2):135–50.

49. Richardson JC, Hassell AB, Hay EM, Thomas E. `I'd rather go and know': women's understanding and experience of DEXA scanning for osteoporosis. Health Expect. 2002 Jun;5(2):114–26.

50. Silverman E, Woloshin S, Schwartz LM, Byram SJ, Welch HG, Fischhoff B. Women's Views on Breast Cancer Risk and Screening Mammography: A Qualitative Interview Study. Med Decis Making. 2001 May;21(3):231–40.

51. Green E, Griffiths F, Thompson D. 'Are My Bones Normal Doctor?' the Role of Technology in Understanding and Communicating Health Risks for Midlife Women. Sociological Research Online. 2006 Dec;11(4):1–16.

52. Sutkowi-Hemstreet A, Vu M, Harris R, Brewer NT, Dolor RJ, Sheridan SL. Adult Patients' Perspectives on the Benefits and Harms of Overused Screening Tests: a Qualitative Study. Journal of General Internal Medicine. 2015 Nov;30(11):1618–26.

53. Stiggelbout A, Copp T, Jacklyn G, Jansen J, Liefers G-J, McCaffery K, et al. Women's Acceptance of Overdetection in Breast Cancer Screening: Can We Assess Harm-Benefit Tradeoffs? Medical Decision Making: An International Journal of the Society for Medical Decision Making. 2020;40(1):42–51.

54. Tacken MAJB, Braspenning JC, Hermens RP, Spreeuwenberg PM, van den Hoogen HJ, de Bakker DH, et al. Uptake of cervical cancer screening in The Netherlands is

For peer review only

mainly influenced by women's beliefs about the screening and by the inviting organization [Internet]. Vol. 17, European journal of public health. Eur J Public Health; 2007 [cited 2020 Oct 14]. Available from: https://pubmed.ncbi.nlm.nih.gov/16837520/

55. Douma LN, Uiters E, Verweij MF, Timmermans DRM. Autonomous and informed decision-making: The case of colorectal cancer screening. PloS One. 2020;15(5):e0233308.

56. Hulme J, Moravac C, Ahmad F, Cleverly S, Lofters A, Ginsburg O, et al. "I want to save my life": Conceptions of cervical and breast cancer screening among urban immigrant women of South Asian and Chinese origin. BMC Public Health [Internet]. 2016 Oct 13 [cited 2020 Nov 5];16. Available from: https://www.ncbi.nlm.nih.gov/pmc/articles/PMC5062908/

57. Hope KA, Moss E, Redman CWE, Sherman SM. Psycho-social influences upon older women's decision to attend cervical screening: A review of current evidence. Prev Med. 2017 Aug;101:60–6.

58. Armstrong N. Navigating the uncertainties of screening: the contribution of social theory. Soc Theory Health. 2019 Jun 1;17(2):158–71.

59. Griffiths F, Bendelow G, Green E, Palmer J. Screening for breast cancer: Medicalization, visualization and the embodied experience: Health: [Internet]. 2010 Oct 25 [cited 2020 Oct 14]; Available from: https://journals.sagepub.com/doi/10.1177/1363459310361599?url_ver=Z39.88-2003&rfr_id=ori%3Arid%3Acrossref.org&rfr_dat=cr_pub++0pubmed

60. Bikker AP, Macdonald S, Robb KA, Conway E, Browne S, Campbell C, et al. Perceived colorectal cancer candidacy and the role of candidacy in colorectal cancer screening. Health, Risk & Society. 2019 Nov 17;21(7–8):352–72.

61. Llanwarne N, Newbould J, Burt J, Campbell JL, Roland M. Wasting the doctor's time? A video-elicitation interview study with patients in primary care. Social Science & Medicine. 2017 Mar 1;176:113–22.

62. Gillespie C. The experience of risk as 'measured vulnerability': health screening and lay uses of numerical risk. Sociology of Health & Illness. 2012;34(2):194–207.

63. Deaton C, Dunbar SB, Moloney M, Sears SF, Ujhelyi MR. Patient experiences with atrial fibrillation and treatment with implantable atrial defibrillation therapy. Heart & Lung. 2003 Sep 1;32(5):291–9.

64. Zweiker D, Zweiker R, Winkler E, Roesch K, Schumacher M, Stepan V, et al. Association between subjective risk perception and objective risk estimation in patients with atrial fibrillation: a cross-sectional study. BMJ Open [Internet]. 2017 Sep 25 [cited 2020 Oct 6];7(9). Available from: https://www.ncbi.nlm.nih.gov/pmc/articles/PMC5623506/

65. Kaufman BG, Kim S, Pieper K, Allen LA, Gersh BJ, Naccarelli GV, et al. Disease understanding in patients newly diagnosed with atrial fibrillation. Heart. 2018 Mar;104(6):494–501.

66. Barnett KN, Weller D, Smith S, Steele RJ, Vedsted P, Orbell S, et al. The contribution of a negative colorectal screening test result to symptom appraisal and help-seeking

For peer review only

behaviour among patients subsequently diagnosed with an interval colorectal cancer. Health Expectations. 2018;21(4):764–73.

67. Deutekom M, Vansenne F, McCaffery K, Essink-Bot M-L, Stronks K, Bossuyt PMM. The effects of screening on health behaviour: a summary of the results of randomized controlled trials. J Public Health (Oxf). 2011 Mar 1;33(1):71–9.

68. Tymstra T, Bieleman B. The Psychosocial Impact of Mass Screening for Cardiovascular Risk Factors. Fam Pract. 1987 Dec 1;4(4):287–90.

69. Solbjør M, Skolbekken J-A, Sætnan AR, Hagen AI, Forsmo S. Could screening participation bias symptom interpretation? An interview study on women's interpretations of and responses to cancer symptoms between mammography screening rounds. BMJ Open. 2012 Jan 1;2(6):e001508.

For peer review only

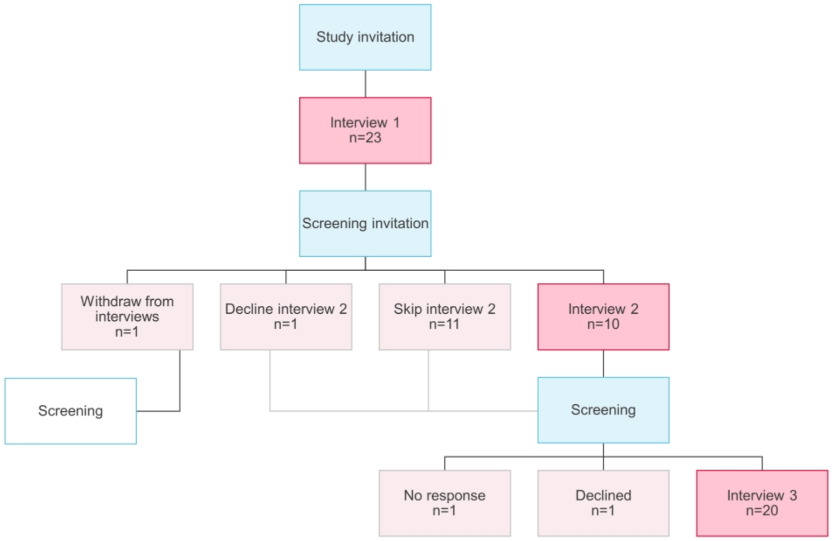

Figure 1: Flowchart of patient interviews

338x190mm (300 x 300 DPI)

COREQ (COnsolidated criteria for REporting Qualitative research) Checklist

A checklist of items that should be included in reports of qualitative research. You must report the page number in your manuscript where you consider each of the items listed in this checklist. If you have not included this information, either revise your manuscript accordingly before submitting or note N/A.

Topic	Item No.	Guide Questions/Description	Reported on Page No.
**Domain 1: Research team and reflexivity**			
Personal characteristics			
Interviewer/facilitator	1	Which author/s conducted the interview or focus group?	
Credentials	2	What were the researcher's credentials? E.g. PhD, MD	
Occupation	3	What was their occupation at the time of the study?	
Gender	4	Was the researcher male or female?	
Experience and training	5	What experience or training did the researcher have?	
Relationship with participants			
Relationship established	6	Was a relationship established prior to study commencement?	
Participant knowledge of the interviewer	7	What did the participants know about the researcher? e.g. personal goals, reasons for doing the research	
Interviewer characteristics	8	What characteristics were reported about the inter viewer/facilitator? e.g. Bias, assumptions, reasons and interests in the research topic	
**Domain 2: Study design**			
Theoretical framework			
Methodological orientation and Theory	9	What methodological orientation was stated to underpin the study? e.g. grounded theory, discourse analysis, ethnography, phenomenology, content analysis	
Participant selection			
Sampling	10	How were participants selected? e.g. purposive, convenience, consecutive, snowball	
Method of approach	11	How were participants approached? e.g. face-to-face, telephone, mail, email	
Sample size	12	How many participants were in the study?	
Non-participation	13	How many people refused to participate or dropped out? Reasons?	
Setting			
Setting of data collection	14	Where was the data collected? e.g. home, clinic, workplace	
Presence of non-participants	15	Was anyone else present besides the participants and researchers?	
Description of sample	16	What are the important characteristics of the sample? e.g. demographic data, date	
Data collection			
Interview guide	17	Were questions, prompts, guides provided by the authors? Was it pilot tested?	
Repeat interviews	18	Were repeat inter views carried out? If yes, how many?	
Audio/visual recording	19	Did the research use audio or visual recording to collect the data?	
Field notes	20	Were field notes made during and/or after the inter view or focus group?	
Duration	21	What was the duration of the inter views or focus group?	
Data saturation	22	Was data saturation discussed?	
Transcripts returned	23	Were transcripts returned to participants for comment and/or	

For peer review only

Topic	Item No.	Guide Questions/Description	Reported on Page No.
		correction?	
**Domain 3: analysis and findings**			
Data analysis			
Number of data coders	24	How many data coders coded the data?	
Description of the coding tree	25	Did authors provide a description of the coding tree?	
Derivation of themes	26	Were themes identified in advance or derived from the data?	
Software	27	What software, if applicable, was used to manage the data?	
Participant checking	28	Did participants provide feedback on the findings?	
Reporting			
Quotations presented	29	Were participant quotations presented to illustrate the themes/findings? Was each quotation identified? e.g. participant number	
Data and findings consistent	30	Was there consistency between the data presented and the findings?	
Clarity of major themes	31	Were major themes clearly presented in the findings?	
Clarity of minor themes	32	Is there a description of diverse cases or discussion of minor themes?	

Developed from: Tong A, Sainsbury P, Craig J. Consolidated criteria for reporting qualitative research (COREQ): a 32-item checklist for interviews and focus groups. *International Journal for Quality in Health Care*. 2007. Volume 19, Number 6: pp. 349 – 357

Once you have completed this checklist, please save a copy and upload it as part of your submission. DO NOT include this checklist as part of the main manuscript document. It must be uploaded as a separate file.

For peer review only

BMJ Open

Why do people take part in atrial fibrillation screening? Qualitative interview study in English primary care

Journal:	*BMJ Open*
Manuscript ID	bmjopen-2021-051703.R1
Article Type:	Original research
Date Submitted by the Author:	21-Oct-2021
Complete List of Authors:	Hoare, Sarah; The Healthcare Improvement Studies Institute, Department of Public Health and Primary Care, University of Cambridge<br>Powell, Alison; The Healthcare Improvement Studies Institute, Department of Public Health and Primary Care, University of Cambridge<br>Modi, Rakesh; University of Cambridge Primary Care Unit, Department of Public Health and Primary Care, University of Cambridge<br>Armstrong, Natalie; University of Leicester, Department of Health Sciences<br>Griffin, Simon; University of Cambridge Primary Care Unit, Department of Public Health and Primary Care, University of Cambridge<br>Mant, Jonathan; University of Cambridge Primary Care Unit, Department of Public Health and Primary Care, University of Cambridge<br>Burt, Jenni; The Healthcare Improvement Studies Institute, Department of Public Health and Primary Care, University of Cambridge<br>SAFER Authorship Group, The; SAFER Programme
**Primary Subject Heading**:	Public health
Secondary Subject Heading:	General practice / Family practice, Cardiovascular medicine, Public health, Health services research
Keywords:	PRIMARY CARE, CARDIOLOGY, PUBLIC HEALTH, QUALITATIVE RESEARCH

SCHOLARONE™
Manuscripts

I, the Submitting Author has the right to grant and does grant on behalf of all authors of the Work (as defined in the below author licence), an exclusive licence and/or a non-exclusive licence for contributions from authors who are: i) UK Crown employees; ii) where BMJ has agreed a CC-BY licence shall apply, and/or iii) in accordance with the terms applicable for US Federal Government officers or employees acting as part of their official duties; on a worldwide, perpetual, irrevocable, royalty-free basis to BMJ Publishing Group Ltd ("BMJ") its licensees and where the relevant Journal is co-owned by BMJ to the co-owners of the Journal, to publish the Work in this journal and any other BMJ products and to exploit all rights, as set out in our licence.

The Submitting Author accepts and understands that any supply made under these terms is made by BMJ to the Submitting Author unless you are acting as an employee on behalf of your employer or a postgraduate student of an affiliated institution which is paying any applicable article publishing charge ("APC") for Open Access articles. Where the Submitting Author wishes to make the Work available on an Open Access basis (and intends to pay the relevant APC), the terms of reuse of such Open Access shall be governed by a Creative Commons licence – details of these licences and which Creative Commons licence will apply to this Work are set out in our licence referred to above.

Other than as permitted in any relevant BMJ Author's Self Archiving Policies, I confirm this Work has not been accepted for publication elsewhere, is not being considered for publication elsewhere and does not duplicate material already published. I confirm all authors consent to publication of this Work and authorise the granting of this licence.

For peer review only

Why do people take part in atrial fibrillation screening? Qualitative interview study in English primary care

Sarah Hoare[1] (BA, MPhil, PhD. Research Associate. https://orcid.org/0000-0002-8933-217X)

Alison Powell[1] (MA, PhD. Research Associate. https://orcid.org/0000-0003-2524-5357)

Rakesh Modi[2] (MBBS MA MRCP MRCGP DCH DRCOG MSc. Academic GP and Wellcome Trust PhD Fellow. ORCID: https://orcid.org/0000-0001-9651-6690)

Natalie Armstrong[3] (BA, MSc, PhD. Professor of Healthcare Improvement Research. https://orcid.org/0000-0003-4046-0119)

Simon Griffin[2] (MBBS MSc DM FRCGP FMedSci Professor of General Practice. http://orcid.org/0000-0002-2157-4797)

Jonathan Mant[2] (MD, FFPH. Professor of Primary Care Research. https://orcid.org/0000-0002-9531-0268)

Jenni Burt[1] (PhD. Senior Social Scientist. https://orcid.org/0000-0002-0037-274X)

and The SAFER Authorship Group.

[1]THIS Institute (The Healthcare Improvement Studies Institute), University of Cambridge, Cambridge Biomedical Campus, Clifford Allbutt Building, Cambridge CB2 0AH, UK

[2]Primary Care Unit, Department of Public Health & Primary Care, University of Cambridge School of Clinical Medicine, Strangeways Research Laboratory, University of Cambridge, Worts Causeway, Cambridge CB1 8RN, UK

[3]SAPPHIRE Research Group, Department of Health Sciences, University of Leicester, George Davies Centre, University Road, Leicester LE1 7RH, UK

Correspondence to: Sarah Hoare, THIS Institute, University of Cambridge, Cambridge Biomedical Campus, Clifford Allbutt Building, Cambridge CB2 0AH, UK. Sarah.hoare@thisinstitute.cam.ac.uk

Word count: abstract: 299; manuscript: 4168

Number of references: 74

Number of figures: 1 **Number of tables:** 1

For peer review only

Statements

Acknowledgements

The SAFER Authorship Group includes:

Andrew Dymond, University of Cambridge, UK

Richard Hobbs, University of Oxford, UK

Rachel Johnson, University of Bristol, UK

Richard McManus, University of Oxford, UK

Kate Williams, University of Cambridge, UK

We thank the SAFER participants for their time and for sharing their experiences of taking part in screening. We also thank the GP practices involved and the local National Institute for Health Research Clinical Research Network for supporting this study. We are grateful to the SAFER study team and in particular to Millie Watson for her work in administering these interviews, and to James Brimicombe for managing the electronic SAFER data.

Ethical approval

The study was approved by the London-Central NHS Research Ethics Committee (reference number: 18/LO/2066).

For peer review only

Why do people take part in atrial fibrillation screening? Qualitative interview study in English primary care

For peer review only

Abstract

Objectives: There is insufficient evidence to support national screening programmes for atrial fibrillation (AF). Nevertheless, some practitioners, policymakers and special interest groups have encouraged introduction of opportunistic screening in primary care in order to reduce the incidence of stroke through earlier detection and treatment of AF. The attitudes of the public towards AF screening are unknown. We aimed to explore why AF screening participants took part in the screening.

Design: Semi-structured longitudinal interview study of participant engagement in the SAFER study (Screening for Atrial Fibrillation with ECG to Reduce stroke). We undertook initial interviews face-to-face, with up to two follow-up telephone interviews during the screening process. We thematically analysed and synthesised this data to understand shared views of screening participation.

Setting: 5 primary care practices in the East of England, UK.

Participants: 23 people (n=53 interviews) taking part in the SAFER study first feasibility phase.

Results: Participants were supportive of screening for AF, explaining their participation in screening as a 'good thing to do'. Participants suggested screening could facilitate earlier diagnosis, more effective treatment, and a better future outcome, despite most being unfamiliar with AF. Participating in AF screening helped attenuate participants' concerns about stroke and demonstrated their commitment to self-care and being a 'good patient'. Participants felt that the screening test was non-invasive, and they were unlikely to have AF; they therefore considered engaging in AF screening was low risk, with few perceived harms.

Conclusions: Participants assessed the SAFER AF screening programme to be a legitimate, relevant, and safe screening opportunity, and complied obediently with what they perceived to be a recommendation to take part. Their unreserved acceptance of screening benefit and lack of awareness of potential harms suggests that uptake would be high but reinforces the importance of ensuring participants receive balanced information about AF screening initiatives.

Keywords: screening, qualitative research, cardiovascular disease

For peer review only

Strengths and Limitations:

- Our research adds to the limited evidence base about atrial fibrillation (AF) screening participation: opportunistic AF screening is encouraged in primary care to reduce the incidence of stroke through earlier detection and treatment of AF, despite insufficient evidence currently available to support systematic AF screening.
- We report the views of people taking part in AF screening as part of a research study, limiting the relevance of our findings for understanding public engagement in either opportunistic AF screening conducted as part of routine primary care or potential future systematic AF screening programmes.
- This was a study of people who participated in AF screening and it does not address the views of those who were invited and opted to not take part.
- The lack of ethnic diversity in our sample reduces the utility of the results, particularly because attitudes towards screening and AF are known to differ by ethnic background.
- Our research contributes to social science literature about the public's 'moral obligation' to participate in screening even when both the programme and the condition are largely unfamiliar to participants.

For peer review only

I. Introduction

Opinion on whether there should be a screening programme for Atrial Fibrillation (AF) is divided [1–3]. Screening advocates include clinical societies [4–7], patient associations [8], clinicians [9,10], and pharmaceutical and technology companies [11,12]. Justifications for AF screening are well-rehearsed. Having AF (a common, often asymptomatic, heart arrhythmia) increases the risk of stroke five-fold [13] with AF-related stroke typically more severe than non-AF-related stroke [14]. AF anticoagulant treatment is effective at reducing stroke risk [15], and screening devices for use in the community are inexpensive [2]. Conversely, caution around AF screening is attributed to insufficient evidence on effectiveness, cost-effectiveness and potential harms of national systematic AF screening programmes, which are not currently recommended [16,17]. Questions remain, for example, about the difference in stroke risk and anticoagulation benefit for people with AF detected by screening rather than in routine care [16,18].

In the meantime, opportunistic AF screening is encouraged in primary care, leading to accusations of 'back-door' screening [12]. Opportunistic screening occurs through many routes. In the UK, clinicians are recommended to assess patients' pulse rhythm as part of NHS health checks [19], driven by an explicit aim of NHS programmes and policy to increase identification and diagnosis of previously undetected AF [20,21]. NHS England has distributed digital ECG devices to increase AF detection [22], and some clinical commissioning groups have encouraged opportunistic AF case-finding in primary care [23,24].

The public's views have largely been absent from this debate. Patient associations which champion AF screening provide important information from the perspective of a motivated minority, but tell us little about the opinions of the wider public [12]. If typically positive attitudes to screening programmes apply here [25,26], it seems likely that the public will be enthusiastic about AF screening, as seen in a small-scale study of AF screening trial participants [27] and informal reports from a previous AF screening trial [28]. However, little is known about the reasons why the public are motivated to participate in AF screening [27,28]. We aimed to explore why participants in SAFER, an AF screening study, opted to take part.

II. Methods

A. Design and participants

Our data are from a longitudinal interview study with participants in the AF screening study SAFER (Screening for Atrial Fibrillation using ECG to Reduce stroke) (https://www.safer.phpc.cam.ac.uk/). We interviewed participants during their study involvement to explore their experience of AF screening, including their views on why they had taken part.

The SAFER programme is ongoing, and interviews were conducted as part of a feasibility study. Participants were drawn from five participating GP (General Practitioner) practices in the East of England; eligible patients were aged 65 years and over, not taking anticoagulant medication, and were neither on a palliative care register nor living in a residential (care) home. Participants were first invited to contribute to SAFER research and subsequently to take part in AF screening. The screening involved using a Zenicor (www.zenicor.com) hand-

For peer review only

held single-lead electrocardiogram (ECG) device four times a day for between one and four weeks.

We selected interview participants from the first wave of feasibility phase participants. Potential interviewees were purposively sampled by age, gender and GP practice to ensure a varied sample. All participants were invited by letter and agreed to take part in SAFER and, on invitation to screening, elected to take part.

B. Data collection

We interviewed participants up to three times throughout their screening. Interview one occurred shortly after participants agreed to take part in the SAFER research study; interview two after a participant had received their invitation to take part in SAFER screening; and interview three after they had completed the screening. Interview one was held either at the participant's home or at their GP practice, and interviews two and three were conducted by telephone. Not all participants took part in all interviews: participants could elect not to take part in each interview phase, and some participants were not invited to the second interview to ensure that they had the opportunity to participate in screening (see Figure 1). Interviews were typically held with only the interviewer ([author 1]) and interview participant, but when participants' partners were present they were included in the discussion and consented to take part. Fieldnotes were completed following each interview.

Interviews were semi-structured and used a flexible topic guide exploring experiences of, and attitudes towards, screening in general and AF screening in particular (see supplemental material).The topic guide was designed to reflect our research question and was refined and iteratively adapted as interviews progressed to accommodate areas of interest raised by participants. Consent was taken prior to each interview. Interviews were conducted in 2019 and continued until we had reached sufficient information power [29] to have a meaningful dataset. Interviews lasted on average 30 minutes each (range 10-90 minutes) and were audio-recorded.

C. Analysis

Interviews were transcribed verbatim and analysed using a thematic approach to explore the reasons why people took part in AF screening. The initial analysis phase focused on 16 transcripts selected for their relevance to addressing the question and drawn from all three interview time-points (interview 1, 7; interview 3, 3; interview 3, 6), followed by analysis of the whole interview dataset. SH conducted coding, on paper and then supported by the software NVivo 12, with codes generated inductively from topics raised by participants and interview fieldnotes, and deductively from the interview schedule. We developed key themes through consensus meetings between SH, AP and JB, and SH then explored these themes within the wider dataset to establish the veracity of key themes and identify deviant cases, with the themes subsequently refined. These themes were synthesised to understand shared views of screening participation, aided by reference to social science and health screening literature about participation in screening.

[Figure 1: Flowchart of interviews in the screening process]

D. Patient and public involvement (PPI)

The SAFER programme is guided by four PPI representatives [30] who have been involved since the programme was conceived and funded. The representatives are embedded within the team (e.g. one is a co-applicant, another sits on the programme steering committee) and advise on all aspects of the design, management and delivery of the programme, including interpreting and disseminating the findings. We have also established a supplementary

qualitative work-stream PPI group with whom we regularly consult about research design questions.

III. Results

We completed 53 interviews with 23 participants (interview 1 n=23, interview 2 n=10, interview 3 n=20). Table 1 lists participants' characteristics. Quotations in the text are followed by the participant's interview ID number (1-53), practice code (A-E) and interview phase (1-3).

[Table 1: Socio-demographics of participants]

A. Summary

Engagement with AF screening within the SAFER study was driven by several inter-connected considerations, many relating to wider attitudes that screening is a 'good thing' to participate in, and to the perceived importance of preventive health behaviours. Participants knew little about AF, but were motivated to be screened by the perceived potential for reducing their risk of catastrophic disability associated with stroke, and a seemingly low-risk screening test with few if any apparent harms and significant perceived benefits.

B. The importance of screening

Participants were familiar with national screening programmes, often referring to their own or a relative's prior experience of taking part in screening. Screening was described as a "no-brainer" by several participants, and it was clear within our sample that screening was seen as a routine healthcare activity in which participation was the only reasonable option. This option was driven by a desire to follow clinical recommendations, gain insight into health status and, if required, access early treatment, and was further underpinned by a 'fear of missing out' by not participating.

1. Clinically recommended screening

Screening invitations were typically seen as legitimate requests from a clinical authority which was presumed to recommend the screening programme. Participants explained that this meant that screening was something they ought to at least consider engaging with, and for a minority as a mandate that they must comply with.

> *"And I assume when something like that [a screening invitation] comes from the post, I immediately comply, I don't question. I don't question because it's come from a…you know, it's a bit different to somebody trying to ring me up and telling me that they can spend my pension more successfully than I can. It comes from an authoritative source, and I don't think there's any question of me not believing what the prospects are." (05B_1)*

2. Reassurance of screening

Participants expected screening would provide them with reassurance about their health. This could be definitive, with a negative test result providing "peace of mind" that the screened for condition was "something else you can tick off the list of things to worry about" (06E_1). Reassurance could also be more anticipatory, whereby participation in screening was protective of regret if in the future one did indeed get diagnosed with the condition but had chosen not to be screened. Simply attending screening also seemed to confer reassurance about healthiness, whereby participants additionally acknowledged responsibility for maintaining their health and preventing ill-health:

For peer review only

"But, I think, it's a brilliant idea and the thing is though, that things can happen in between, so you've still got to be responsible for your own health."
(25A_1)

3. Early diagnosis, early treatment, better outcomes

Participants were clear that early identification of a screened condition would enable preventive action or prompt treatment to avert ill-health or reduce disease severity. Participants placed different emphases on the importance of these potentialities (from prevention to treatment) but were united by a presumption that earliness was advantageous. By comparison, 'late' identification or treatment was associated with perceived poorer outcomes for individuals and the healthcare system. Participants often drew on this binary conceptualisation of early versus late identification to explain why it was sensible to take part in screening:

"I'd rather find something out, if there is a screening thing that can indicate early symptoms that can be caught early and something can be done about it, I think I'd much rather have that option than sort of find out later, you know, further down the road that, well it's too late now." (16D_1)

a) Identifying silent conditions: robust test

Underlying perceived benefits of early diagnosis was participants' awareness that screening could identify a condition before symptoms could be noticed or manifested, or for which symptoms were absent (a 'silent' condition):

"I get this thing for the bowel screening, which isn't as much fun. And despite that, I've gone in for it, because as a layman, I simply have no idea what's going on inside my body. The fact I feel fit, doesn't mean I am fit, you know, from those sort of complaints. [I: So…] So the answer is, I've said yes to that [bowel cancer screening] and I would have said yes to this [AF screening]."
(11A_2)

The ability of screening to identify silent conditions led to a view amongst some participants that screening tests could provide a particularly in-depth or robust review of health. For a few this extended the scope beyond the screened condition; here, the SAFER AF screening came to be viewed as a "free medical" (15A_3). Screening thus offered ready access to healthcare and perceived "health checks" without the need to present tangible health symptoms:

R: "I think, I don't want to bother the doctor he's so busy, so I won't bother to go until I really think, I'm going to have to go. […] I think, a lot of people don't go, so screening, perhaps, would ensure that they are seen for a particular thing, once every three years or however they're done."

I: "And, do you think going to a screening appointment is different to just going for something else?"

R: "Well, I think, it has to be 'cause I don't think anybody, unless they've got any symptoms, has any reason to think they've got anything."
(25A_1)

b) Not taking part

Associated with reducing the risk of 'late' diagnosis, participating in screening also protected participants from moral judgement about *not* engaging in screening. Non-screeners were

For peer review only

variously portrayed by participants as unduly embarrassed, uninformed, indolent, irresponsible, wilfully ignorant, or gratuitously anxious, and often a combination of these. A significant part of participants' moralising about non-screeners was concern that they were missing out on the benefit of screening:

> *"I mean maybe there are some people that don't want to know. They live in a dream world and…but I just think if you've got the chance to do something about it and it gives you a better quality, even if it's only a short one, that's great, you should grab it."*
> *(15A_3)*

C. AF screening

1. Views about AF

AF was a new condition to most participants, except those with first-hand experience as a result of a family member's diagnosis or prior clinical knowledge. Some participants struggled to pronounce the term and many explained that they had conducted internet research to better understand the condition, supplementing the study literature provided. Despite this, after interviewer explanation, participants engaged with the concept of an irregular heartbeat and described what they thought it might be like to have the condition. At best, participants understood AF as a benign condition, but most expressed concern about the association with stroke. Participants anticipated that, if they had AF, they would worry about having a major and sudden heart-related incident, and expected having to modify their lifestyle (for some to a very significant and limiting degree) and to receive corrective treatment to address it:

> *I: […] "I wonder what you thought it might be like to have an irregular heartbeat?"*
>
> *R: "A little bit disconcerting. I would say you would want it regulated, maybe if it's to have a pacemaker fitted or something. You wouldn't want to live with something that you know…you'd probably be able to feel it beating faster. You wouldn't want to think to yourself, am I having a stroke shortly? So I would say it's more peace of mind to know that you can regulate it if you have a… I mean, if you know that your heart is beating faster and it could be a start of a stroke, and you can take a pill to stop it and you didn't know about it, you'd be a bit miffed to say if you didn't have one."*
> *(06E_1)*

2. Views about AF screening

Most participants did not expect to receive a diagnosis of AF as a result of taking up the offer of screening. They explained this in relation to their own perception of their health, whether because they were healthy overall, or because they identified an absence of AF symptoms or prior heart-related issues. Despite this, participants explained that participating in screening was useful because there was a *chance* that they may unknowingly have AF. As a silent condition with perceived serious consequences and a presumption for curative treatment, participants recognised the utility of checking to see if they had it:

> *I: "Do you think the atrial fibrillation screening is relevant to you?"*
>
> *R: "I don't suffer from any of those symptoms. That's as far as I can…or I'm not aware of suffering from those symptoms. That's as far as I can go. Like a lot of things that are more evident with age, I think it ill behoves me*

For peer review only

to say, oh, I'm, you know, I don't need to be tested because I feel fine."
(11A_2)

3. Stroke fear

Participants were clear that stroke was not a condition they wished to experience. The consequences of stroke were recognised to be potentially debilitating and life-changing. Whilst those who knew people who had had a stroke often recognised a diversity of recovery experiences that included more positive outcomes, there remained a concern about the high level of disability stroke could cause.

Participants typically recognised both that a stroke could happen unexpectedly "to anybody at any time, any day, any age really" (16D_1) and that there were genetic and modifiable lifestyle factors which could increase the risk of having a stroke. Together with concern about the consequences of having a stroke, this dual conception of stroke as preventable and unexpected made the association between AF and stroke significant, and thus AF screening particularly worthwhile:

I: "What do you think about the link between atrial fibrillation and stroke?"

R: "I would try to do everything I can not to have a stroke. So if there was something I could do that would stop me turning into a vegetable, I will do it. So as far as I'm concerned, it's a no-brainer. Once you've had the stroke, whatever life you have got left, it's not going to be much fun, so try and avoid it."
(06E_1)

4. Low-risk screening

The AF screening test involved participants placing their thumbs or two fingers on a portable ECG device. Participants unanimously recognised this to be non-invasive and typically understood the screening to be safe and to not cause harm, often aligning it with the ease of measuring blood pressure.

Participants acknowledged that anxiety around the screening test may arise for others. However, they frequently related this to the screened individuals' psychological state rather than something inherent to the testing process. Participants rarely recognised other iatrogenic screening harms. If they did, such harms were presented as legitimate concerns and often as intrinsic to testing. However, participants presumed the screening was safe and, when discussed, positioned consideration of these issues as the responsibility of the screening provider.

Compared to the perceived fuzzy and inconsequential harms of screening, participants saw the advantages of AF screening as tangible and significant. Screening could identify AF 'early', using a test that was perceived to cause no harm, and which could ameliorate the risk of stroke:

R1: "Any harm? No I don't think that any harm can arise from that all, personally, screening, no."

R2: "I mean to say, it looks as if just by putting your thumbs on the actual screening test on the box, you're not going to get anything invasive from that. And, I say, if it shows up something that can be dealt with sooner rather than later, that can only be a good thing in our view really."
(16D_2)

For peer review only

IV. Discussion

A. Summary

We explored the reasons why people took part in AF screening through interviews with SAFER study participants. Their accounts presented screening as a routine obligation and something one 'ought' to do as a responsible to ensure good health and prevent illness. Limited awareness of AF did not detract from their view of the utility of AF screening – rather, preventing stroke was a strong rationale for participating. Participants assessed AF screening to be a legitimate, relevant and safe screening opportunity, and complied obediently with what they perceived to be a recommendation to take part.

B. Strengths and limitations

Our research adds to the limited evidence about AF screening participation. Our findings also contribute to social science literature about patients' 'moral obligation' [31] to participate in screening [31–34], even when both the programme and the condition are largely unfamiliar to participants.

It is likely that our results reflect the delivery of SAFER as a research study. Whilst the SAFER study has been designed to mimic a national screening programme, participants were aware that it was research. This may limit the relevance of our findings for understanding public engagement in either opportunistic AF screening conducted as part of routine primary care, or potential future systematic AF screening programmes. For example, participants reported that a key reason for taking part was to 'help out', a prevalent and well-recognised motivation for participating in research [35].

This was a study of people who participated in AF screening, and does not address the views of those who were invited and opted to not take part. Nevertheless, focusing on people who did engage has allowed us to understand the information needs and potential misconceptions of this population.

Participants' familiarity with, and participation in, national screening programmes suggests their enthusiasm for AF screening will not be relevant to all screening invitees. For example, recent studies in the UK found that prior attendance in screening is positively correlated with future participation.[36,37] Participants in our study match national trends by being less deprived by practice deprivation status [38]. The lack of ethnic diversity in our sample will also necessarily reduce the utility of the results, particularly because screening uptake and attitudes towards AF differ by ethnic background [38,39].

C. Comparison with existing literature

Participants' concern about having a stroke mediated their unfamiliarity with AF and encouraged them to take part. The novelty of AF is unsurprising as studies find limited awareness of the condition, whether the participants' relationship to AF is: just at risk [40]; being screened for it [27]; prior to diagnosis [41]; or having it [39,42]. Similarly, those with AF report being worried or fearful about their condition [41,42], consider it a serious heart disease [43] and overestimate the risk of stroke [44]. AF was explained to participants in the context of stroke-risk, and this may explain participants' worries about AF-induced stroke: the majority of AF patients in a 2002 study [39] did not consider their condition to be severe, but almost half were not aware that having AF predisposed them to stroke.

The enthusiasm participants had for AF screening, and the weighting they attributed to the benefits versus the harms of screening, accord with other studies on the public's experience

For peer review only

of national screening programmes [45,46]. There is widespread public support for screening even in survey scenarios in which there was no treatment for the screened condition [25,26], or if screening is not clinically recommended because participants are outside screening age thresholds [47,48]. The benefits SAFER participants attributed to AF screening concur with reported experience. Screening offers the hope of reassurance [31,49,50] and confirmation of healthiness [49], or in the worst case, the benefit in knowing one's ill-health status [51] and associated advantageous early diagnosis or treatment [52]. Participation also offers an 'insurance policy' [53], whereby the often presumed 'health check' [50] means those taking part can be assured that 'they had done everything they could' [49] if they later develop the screened condition. Like SAFER participants, this enthusiasm is contextualised by reported limited concern or awareness of screening harms [52]. A recent US study found that many respondents could not name any harms of screening, and where they could, focused, as SAFER participants and other AF screening participants did [27], on the direct harms of the screening test itself [54]. Even when harms are recognised, these are typically of a lower priority to participants compared to the benefits of screening [55].

Informing participants' consideration of the harms and benefits of screening was an assumption that taking part in screening was something they ought to do, corresponding with evidence from screening decision-making studies [48,56–59]. Sociological work has shown how screening participation is associated with maintaining, and being responsible for, one's own health [31–34,49,50,60–62], and part of patients' efforts to be a 'good patient' and to use healthcare resources appropriately [63].

D. Implications for practice and research

The public can be expected to be unfamiliar with AF, to anticipate that screening is recommended, to perceive any AF test as a comprehensive heart function review and to be positive about screening. Clinicians should be prepared to work with members of the public interested in screening to help them understand what they are being offered and what the risks and benefits of taking part are. SAFER participants suggested that early identification leads to better results. Counselling individuals on the benefits and risks of AF screening is particularly pertinent given participant expectations for curative treatment and because clinical evidence on the benefit of managing screen-detected AF is still uncertain [18]. As with all screening, benefits must be weighed against harms [64].

Receiving a positive result may be unexpected for those engaging in screening for health confirmation, and could engender feelings of health vulnerability [65]. These participants may need help to moderate concerns about the severity and lifestyle impact of the condition. Clinician engagement is significant because their reactions and approaches to AF have been found to impact patients' perceptions of their condition [66], and patients' understanding of AF may be aided by often called-for patient education interventions [39,44,67,68]. Negative test results may confer 'healthiness' [69] and induce false reassurance about their stroke risk. Though the evidence-base is small [70] it is plausible that participants' existing 'unhealthy' behaviours are validated as acceptable and continued [71], whilst any future AF-related symptoms may be 'downplayed' [72], discouraging prompt healthcare seeking. Consequently, clinicians are recommended to contextualise negative results with reference to the necessary limitations of any screening test and participants' ongoing risk of developing the condition [69].

Our study has contributed to a small evidence-base on public experience of AF screening [27]. If AF screening is demonstrated to be effective, then complementary studies would be important to understand the attitudes of people who do not participate in screening and how participants (particularly those with positive results) weigh up the benefits and harms throughout the screening process.

V. Tables

Table 1: Socio-demographics of participants (n=23)

Participant characteristic	*n*
**Age group**	
65-69 years	9
70-74 years	7
75-79 years	5
80+ years	2
**Sex**	
Female	10
Male	13
**Practice** (*Deprivation score 1-10; 10 = least deprived*)[1]	
A[2] *(10)*	8
B *(8)*	5
C *(8)*	4
D *(6)*	4
E *(5)*	2

[1] The deprivation score is taken from National General Practice Profiles [73], using the English Indices of Deprivation to calculate the Index of Multiple Deprivation, which provides "an overall measure of deprivation experienced by people living in an area" [73,74]
[2] Practice name pseudonym

For peer review only

VI. Contributions

JB planned the interview study and oversaw the data collection by SH. SH led the analysis with themes developed with JB and AP, and contributions from RM. SH and JB drafted the original manuscript. SH, AP, RM, NA, SG, JM, JB critically reviewed, revised, and approved the final version. All members of the SAFER Authorship Group (AD, RH, RJ, RM, KW) reviewed and approved the initial submission and had the opportunity to comment, and the final version was shared with them.

For peer review only

VII. Competing interests

JM has received honoraria from BMS/Pfizer.

VIII. Funding

The SAFER study is funded by the National Institute for Health Research (NIHR) Programme Grants for Applied Research (grant reference number RP-PG-0217-20007) and School for Primary Care Research (SPCR-2014-10043, project 410). The views expressed are those of the authors and not necessarily those of the NIHR or the Department of Health and Social Care.

RM is supported by the Wellcome Trust as part of the Wellcome Trust PhD Programme for Primary Care Clinicians (grant reference number 203921/Z/16/Z).

NA is supported by a Health Foundation Improvement Science Fellowship (N/A award/grant number) and also by the National Institute for Health Research (NIHR) Applied Research Collaboration East Midlands (ARC EM) (N/A award/grant number). The views expressed are those of the authors and not necessarily those of the NHS, the NIHR or the Department of Health and Social Care.

The University of Cambridge has received salary support in respect of SG from the NHS in the East of England through the Clinical Academic Reserve (N/A award/grant number).

JM is an NIHR Senior Investigator (N/A award/grant number).

SH, AP and JB are based in The Healthcare Improvement Studies Institute (THIS Institute), University of Cambridge. THIS Institute is supported by the Health Foundation, an independent charity committed to bringing about better health and healthcare for people in the UK (N/A award/grant number).

Data sharing

The data may be available on request from the authors, subject to appropriate permissions: please email the PI Jenni Burt (jenni.burt@thisinstitute.cam.ac.uk) for details.

IX. References

1. Neubeck L, Orchard J, Lowres N, Freedman SB. To Screen or Not to Screen? Examining the Arguments Against Screening for Atrial Fibrillation. Heart Lung Circ. 2017 Sep;26(9):880–6.

2. Lown M, Garrard J, Irving G, Edwards D, Hobbs FR, Mant J. Should we screen for atrial fibrillation? British Journal of General Practice. 2017 Jul;67(660):296–7.

3. Lown M, Moran P. Should we screen for atrial fibrillation? BMJ. 2019 Feb 13;l43.

4. Hobbs FR, Taylor CJ, Jan Geersing G, Rutten FH, Brouwer JR. European Primary Care Cardiovascular Society (EPCCS) consensus guidance on stroke prevention in atrial fibrillation (SPAF) in primary care. Eur J Prev Cardiol. 2016 Mar;23(5):460–73.

5. Kirchhof P, Benussi S, Kotecha D, Ahlsson A, Atar D, Casadei B, et al. 2016 ESC Guidelines for the management of atrial fibrillation developed in collaboration with EACTS. Eur Heart J. 2016 Oct 7;37(38):2893–962.

6. Lip GY, Ramsay SG. Insights from the RCPE UK Consensus Conference on approaching the comprehensive management of atrial fibrillation. Expert Review of Cardiovascular Therapy. 2012 Jun 1;10(6):697–700.

7. Authors/Task Force Members, Camm AJ, Lip GYH, De Caterina R, Savelieva I, Atar D, et al. 2012 focused update of the ESC Guidelines for the management of atrial fibrillationAn update of the 2010 ESC Guidelines for the management of atrial fibrillation Developed with the special contribution of the European Heart Rhythm Association. Europace. 2012 Aug 24;14(10):1385–413.

8. Atrial Fibrillation Association, AntiCoagulation Europe. The AF Report. Atrial Fibrillation: Preventing A Stroke Crisis [Internet]. Atrial Fibrillation Association and AntiCoagulation Europe; 2011. Available from: http://atrialfibrillationassociation.org.uk/app/webroot/files/file/140508-cw-FINAL-The%20AF%20Report.pdf

9. Robson J, Schilling R. Atrial fibrillation: time for active case finding. British Journal of General Practice. 2019 Feb;69(679):58–9.

10. Kearney M, Fay M, Fitzmaurice DA. Stroke prevention in atrial fibrillation: we can do better. Br J Gen Pract. 2016 Feb 1;66(643):62–3.

11. Mahase E. Atrial fibrillation groups lobby patients to overturn screening committee decision. BMJ [Internet]. 2019 Jul 9 [cited 2020 Oct 2];366. Available from: https://www.bmj.com/content/366/bmj.l4612

12. McCartney M, Fell G, Finnikin S, Hunt H, McHugh M, Gray M. Why 'case finding' is bad science. J R Soc Med. 2019 Dec 12;014107681989142.

13. Wolf PA, Abbott RD, Kannel WB. Atrial fibrillation as an independent risk factor for stroke: the Framingham Study. Stroke. 1991 Aug;22:983–8.

For peer review only

14. Marini C, De Santis F, Sacco S, Russo T, Olivieri L, Totaro R, et al. Contribution of atrial fibrillation to incidence and outcome of ischemic stroke: results from a population-based study. Stroke. 2005 Jun;36:1115–9.

15. G. Hart R, A. Pearce L, I. Aguilar M. Meta-analysis: Antithrombotic Therapy to Prevent Stroke in Patients Who Have Nonvalvular Atrial Fibrillation. Annals of Internal Medicine [Internet]. 2007 Jun 19 [cited 2020 Nov 20]; Available from: https://www-acpjournals-org.ezp.lib.cam.ac.uk/doi/abs/10.7326/0003-4819-146-12-200706190-00007

16. King S, Fitzgerald A, Bartlett C, Mahon J, Arber M, Carr E, et al. Evidence Summary for Screening for Atrial Fibrillation in Adults: External review against programme appraisal criteria for the UK National Screening Committee. London: UK National Screening Committee; 2019.

17. US Preventive Services Task Force, Curry SJ, Krist AH, Owens DK, Barry MJ, Caughey AB, et al. Screening for Atrial Fibrillation With Electrocardiography: US Preventive Services Task Force Recommendation Statement. JAMA. 2018 Aug 7;320(5):478.

18. Jones NR, Taylor CJ, Hobbs FDR, Bowman L, Casadei B. Screening for atrial fibrillation: a call for evidence. Eur Heart J. 2020 Mar 7;41(10):1075–85.

19. NHS Health Check: Best practice guidance [Internet]. London: Public Health England; 2017 [cited 2020 Oct 2]. Available from: https://www.england.nhs.uk/london/wp-content/uploads/sites/8/2019/08/2.1.7-NHS-Health-Check-Best-Practice-Guidance-2017.pdf

20. NHS RightCare » High value intervention in atrial fibrillation [Internet]. [cited 2020 Oct 9]. Available from: https://www.england.nhs.uk/rightcare/products/pathways/cvd-pathway/af/

21. NHS. The NHS Long Term Plan [Internet]. 2019 [cited 2020 Sep 23]. Available from: https://www.longtermplan.nhs.uk/wp-content/uploads/2019/08/nhs-long-term-plan-version-1.2.pdf

22. Independent Evaluation of the AHSN Network mobile ECG rollout programme: Full Report. Southampton: Wessex Academic Health Science Network; 2019 p. 82.

23. Cole J, Torabi P, Dostal I, Homer K, Robson J. Opportunistic pulse checks in primary care to improve recognition of atrial fibrillation: a retrospective analysis of electronic patient records. British Journal of General Practice. 2018 Jun;68(671):e388–93.

24. Smith T, Fell C, Otete H, Chauhan U. GP incentives to design hypertension and atrial fibrillation local quality-improvement schemes: a controlled before–after study in UK primary care. Br J Gen Pract. 2019 Oct 1;69(687):e689–96.

25. Schwartz LM, Woloshin S, Floyd J. Fowler J, Welch HG. Enthusiasm for Cancer Screening in the United States. JAMA. 2004 Jan 7;291(1):71–8.

26. Waller J, Osborne K, Wardle J. Enthusiasm for cancer screening in Great Britain: a general population survey. Br J Cancer. 2015 Feb 3;112(3):562–6.

For peer review only

27. Lown M, Wilcox CR, Hughes S, Santer M, Lewith G, Moore M, et al. Patients' views about screening for atrial fibrillation (AF): a qualitative study in primary care. BMJ Open. 2020 Mar 1;10(3):e033061.

28. Bury G, Swan D, Cullen W, Keane D, Tobin H, Egan M, et al. Screening for atrial fibrillation in general practice: A national, cross-sectional study of an innovative technology. International Journal of Cardiology. 2015 Jan 15;178:247–52.

29. Malterud K, Siersma VD, Guassora AD. Sample Size in Qualitative Interview Studies: Guided by Information Power. Qualitative Health Research. 2016 Nov;26(13):1753–60.

30. Turk A, Boylan A-M, Locock L. A Researcher's Guide to Patient and Public Involvement [Internet]. University of Oxford; [cited 2021 Oct 1]. Available from: https://oxfordbrc.nihr.ac.uk/wp-content/uploads/2017/03/A-Researchers-Guide-to-PPI.pdf

31. Howson A. Cervical screening, compliance and moral obligation. Sociology of Health & Illness. 1999;21(4):401–25.

32. Bush J. "It's just part of being a woman": cervical screening, the body and femininity. Social Science & Medicine. 2000 Feb 1;50(3):429–44.

33. Ward PR, Coffey C, Meyer S. Trust, choice and obligation: a qualitative study of enablers of colorectal cancer screening in South Australia. Sociology of Health & Illness. 2015;37(7):988–1006.

34. Howson A. Embodied obligation: The female body and health surveillance. In: Nettleton S, Watson J, editors. The body in everyday life. ProQuest Ebook Central, http://ebookcentral.proquest.com: Taylor and Francis; 1998.

35. Sheridan R, Martin-Kerry J, Hudson J, Parker A, Bower P, Knapp P. Why do patients take part in research? An overview of systematic reviews of psychosocial barriers and facilitators. Trials [Internet]. 2020 Mar 12 [cited 2020 Oct 21];21. Available from: https://www.ncbi.nlm.nih.gov/pmc/articles/PMC7069042/

36. Rebolj M, Parmar D, Maroni R, Blyuss O, Duffy SW. Concurrent participation in screening for cervical, breast, and bowel cancer in England. J Med Screen. 2020 Mar;27(1):9–17.

37. McCowan C, McSkimming P, Papworth R, Kotzur M, McConnachie A, Macdonald S, et al. Comparing uptake across breast, cervical and bowel screening at an individual level: a retrospective cohort study. British Journal of Cancer. 2019;121(8):710–4.

38. Public Health England. Supporting the health system to reduce inequalities in screening. https://assets.publishing.service.gov.uk/government/uploads/system/uploads/attachment_data/file/816245/PHE_Screening_inequalities_strategy_2018__1_.pdf: Public Health England; 2019 p. 27. Report No.: GW-489.

39. Lip GYH, Kamath S, Jafri M, Mohammed A, Bareford D. Ethnic Differences in Patient Perceptions of Atrial Fibrillation and Anticoagulation Therapy. Stroke. 2002 Jan 1;33(1):238–42.

40. McCabe PJ, Barton DL, DeVon HA. Older Adults at Risk for Atrial Fibrillation Lack Knowledge and Confidence to Seek Treatment for Signs and Symptoms. SAGE Open

For peer review only

Nurs [Internet]. 2017 [cited 2020 Oct 6];3. Available from: https://www.ncbi.nlm.nih.gov/pmc/articles/PMC6326385/

41. McCabe PJ, Rhudy LM, DeVon HA. Patients' experiences from symptom onset to initial treatment for atrial fibrillation. Journal of Clinical Nursing. 2015;24(5–6):786–96.

42. Aliot E, Breithardt G, Brugada J, Camm J, Lip GYH, Vardas PE, et al. An international survey of physician and patient understanding, perception, and attitudes to atrial fibrillation and its contribution to cardiovascular disease morbidity and mortality. Europace. 2010 May;12(5):626–33.

43. Koponen L, Rekola L, Ruotsalainen T, Lehto M, Leino-Kilpi H, Voipio-Pulkki L-M. Patient knowledge of atrial fibrillation: 3-month follow-up after an emergency room visit. Journal of Advanced Nursing. 2008;61(1):51–61.

44. Mehawej J, Saczynski J, Gurwitz JH, Abu HO, Bamgbade BA, WANG W-J, et al. Self-reported risk of stroke and factors associated with underestimation of stroke risk among older adults with atrial fibrillation: the SAGE-AF study. J Geriatr Cardiol. 2020 Aug;17(8):502–9.

45. Hoffmann TC, Del Mar C. Patients' expectations of the benefits and harms of treatments, screening, and tests: a systematic review. JAMA Intern Med. 2015 Feb;175(2):274–86.

46. Gigerenzer G, Mata J, Frank R. Public knowledge of benefits of breast and prostate cancer screening in Europe. Journal of the National Cancer Institute. 2009 Sep 2;101(17):1216–20.

47. Kotwal AA, Walter LC, Lee SJ, Dale W. Are We Choosing Wisely? Older Adults' Cancer Screening Intentions and Recalled Discussions with Physicians About Stopping. Journal of General Internal Medicine. 2019;34(8):1538–45.

48. Torke AM, Schwartz PH, Holtz LR, Montz K, Sachs GA. Older adults and forgoing cancer screening: 'I think it would be strange'. JAMA internal medicine. 2013 Apr 8;173(7):526–31.

49. Willis K. Personal choice/social responsibility: Women aged 40–49 years and mammography screening. Journal of Sociology. 2004 Jun;40(2):121–36.

50. Willis K. "I Come Because I Am Called": Recruitment and Participation in Mammography Screening in Uppsala, Sweden. Health Care for Women International. 2008 Feb 11;29(2):135–50.

51. Richardson JC, Hassell AB, Hay EM, Thomas E. `I'd rather go and know': women's understanding and experience of DEXA scanning for osteoporosis. Health Expect. 2002 Jun;5(2):114–26.

52. Silverman E, Woloshin S, Schwartz LM, Byram SJ, Welch HG, Fischhoff B. Women's Views on Breast Cancer Risk and Screening Mammography: A Qualitative Interview Study. Med Decis Making. 2001 May;21(3):231–40.

53. Green E, Griffiths F, Thompson D. 'Are My Bones Normal Doctor?' the Role of Technology in Understanding and Communicating Health Risks for Midlife Women. Sociological Research Online. 2006 Dec;11(4):1–16.

For peer review only

54. Sutkowi-Hemstreet A, Vu M, Harris R, Brewer NT, Dolor RJ, Sheridan SL. Adult Patients' Perspectives on the Benefits and Harms of Overused Screening Tests: a Qualitative Study. Journal of General Internal Medicine. 2015 Nov;30(11):1618–26.

55. Stiggelbout A, Copp T, Jacklyn G, Jansen J, Liefers G-J, McCaffery K, et al. Women's Acceptance of Overdetection in Breast Cancer Screening: Can We Assess Harm-Benefit Tradeoffs? Medical Decision Making: An International Journal of the Society for Medical Decision Making. 2020;40(1):42–51.

56. Tacken MAJB, Braspenning JC, Hermens RP, Spreeuwenberg PM, van den Hoogen HJ, de Bakker DH, et al. Uptake of cervical cancer screening in The Netherlands is mainly influenced by women's beliefs about the screening and by the inviting organization [Internet]. Vol. 17, European journal of public health. Eur J Public Health; 2007 [cited 2020 Oct 14]. Available from: https://pubmed.ncbi.nlm.nih.gov/16837520/

57. Douma LN, Uiters E, Verweij MF, Timmermans DRM. Autonomous and informed decision-making: The case of colorectal cancer screening. PloS One. 2020;15(5):e0233308.

58. Hulme J, Moravac C, Ahmad F, Cleverly S, Lofters A, Ginsburg O, et al. "I want to save my life": Conceptions of cervical and breast cancer screening among urban immigrant women of South Asian and Chinese origin. BMC Public Health [Internet]. 2016 Oct 13 [cited 2020 Nov 5];16. Available from: https://www.ncbi.nlm.nih.gov/pmc/articles/PMC5062908/

59. Hope KA, Moss E, Redman CWE, Sherman SM. Psycho-social influences upon older women's decision to attend cervical screening: A review of current evidence. Prev Med. 2017 Aug;101:60–6.

60. Armstrong N. Navigating the uncertainties of screening: the contribution of social theory. Soc Theory Health. 2019 Jun 1;17(2):158–71.

61. Griffiths F, Bendelow G, Green E, Palmer J. Screening for breast cancer: Medicalization, visualization and the embodied experience: Health: [Internet]. 2010 Oct 25 [cited 2020 Oct 14]; Available from: https://journals.sagepub.com/doi/10.1177/1363459310361599?url_ver=Z39.88-2003&rfr_id=ori%3Arid%3Acrossref.org&rfr_dat=cr_pub++0pubmed

62. Bikker AP, Macdonald S, Robb KA, Conway E, Browne S, Campbell C, et al. Perceived colorectal cancer candidacy and the role of candidacy in colorectal cancer screening. Health, Risk & Society. 2019 Nov 17;21(7–8):352–72.

63. Llanwarne N, Newbould J, Burt J, Campbell JL, Roland M. Wasting the doctor's time? A video-elicitation interview study with patients in primary care. Social Science & Medicine. 2017 Mar 1;176:113–22.

64. Angela E Raffle, Mackie A, Gray JAM. Screening: Evidence and Practice. Second edition. Oxford: Oxford University Press;

65. Gillespie C. The experience of risk as 'measured vulnerability': health screening and lay uses of numerical risk. Sociology of Health & Illness. 2012;34(2):194–207.

66. Deaton C, Dunbar SB, Moloney M, Sears SF, Ujhelyi MR. Patient experiences with atrial fibrillation and treatment with implantable atrial defibrillation therapy. Heart & Lung. 2003 Sep 1;32(5):291–9.

For peer review only

67. Zweiker D, Zweiker R, Winkler E, Roesch K, Schumacher M, Stepan V, et al. Association between subjective risk perception and objective risk estimation in patients with atrial fibrillation: a cross-sectional study. BMJ Open [Internet]. 2017 Sep 25 [cited 2020 Oct 6];7(9). Available from: https://www.ncbi.nlm.nih.gov/pmc/articles/PMC5623506/

68. Kaufman BG, Kim S, Pieper K, Allen LA, Gersh BJ, Naccarelli GV, et al. Disease understanding in patients newly diagnosed with atrial fibrillation. Heart. 2018 Mar;104(6):494–501.

69. Barnett KN, Weller D, Smith S, Steele RJ, Vedsted P, Orbell S, et al. The contribution of a negative colorectal screening test result to symptom appraisal and help-seeking behaviour among patients subsequently diagnosed with an interval colorectal cancer. Health Expectations. 2018;21(4):764–73.

70. Deutekom M, Vansenne F, McCaffery K, Essink-Bot M-L, Stronks K, Bossuyt PMM. The effects of screening on health behaviour: a summary of the results of randomized controlled trials. J Public Health (Oxf). 2011 Mar 1;33(1):71–9.

71. Tymstra T, Bieleman B. The Psychosocial Impact of Mass Screening for Cardiovascular Risk Factors. Fam Pract. 1987 Dec 1;4(4):287–90.

72. Solbjør M, Skolbekken J-A, Sætnan AR, Hagen AI, Forsmo S. Could screening participation bias symptom interpretation? An interview study on women's interpretations of and responses to cancer symptoms between mammography screening rounds. BMJ Open. 2012 Jan 1;2(6):e001508.

73. Public Health England. Public Health Profiles [Internet]. [cited 2021 Oct 5]. Available from: https://fingertips.phe.org.uk/

74. National Statistics. English indices of deprivation 2019 [Internet]. GOV.UK. [cited 2021 Oct 5]. Available from: https://www.gov.uk/government/statistics/english-indices-of-deprivation-2019

For peer review only

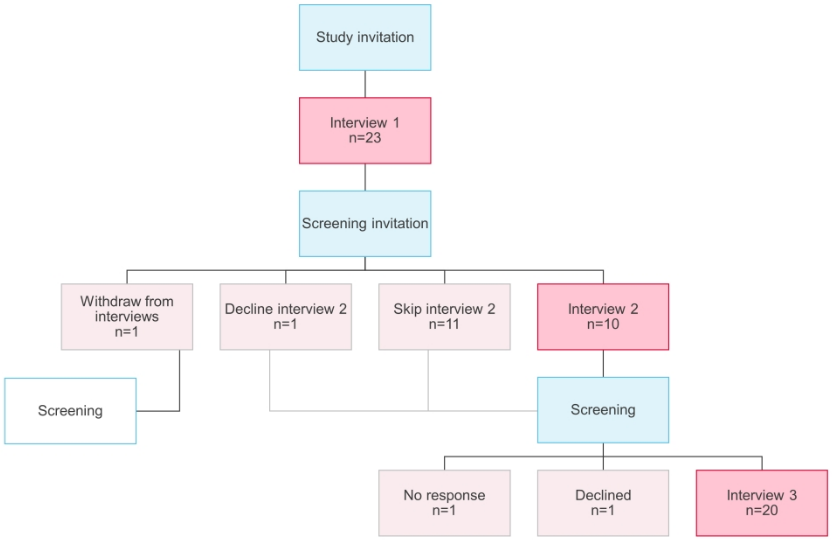

Figure 1: Flowchart of interviews in the screening process

338x190mm (300 x 300 DPI)

SAFER study patient interview topic guide

Introduction and Consent Process

- Researcher to introduce themselves and check understanding of study and processes
- Informed consent and permission to audio record the interview obtained from the participant and partner (or other) if present
- (If applicable) - Explain to patient that this interview is like the previous interview they completed but will now focus on either their views about the screening invitation or their experience of screening.
- Select schedule based on time point of interview.

Interview 1 (post SAFER study invitation)

A. Experiences of screening

I'd like to start by thinking quite generally about screening programmes. There are national screening programmes for many different conditions – things like breast cancer, bowel cancer, and abdominal aortic aneurysm screening. What do you think about screening programmes?

Prompts:

- *Why do you think screening programmes are offered by the NHS?*
- *Potential benefits of screening*
- *Potential harms of screening*

Have you had experience of one of these screening programmes? I'm not asking for you to reveal personal information, just wanting to see if you had decided to go along to screening for something in response to an invitation.

Prompts:

- *Positive or negative experiences [if seemingly acceptable]*

B. Focus on atrial fibrillation

OK, we are going to move on to thinking about this particular condition – atrial fibrillation.

Had you ever heard of atrial fibrillation before you received the study invite? I'm not expecting you to know anything about it, just wanted to check in in case you do.

Prompts:

- *Does anyone they know have AF?*
- *Have they heard anything about AF from any other source?*

[Researcher to explain about atrial fibrillation]:

"Atrial fibrillation (AF) is a condition where the heart has an irregular beat. It may not cause symptoms, occurs in up to 10% of people over 65 years old. AF greatly increases risk of stroke by increasing the chance of blood clots developing. However, there is treatment that inhibits blood clotting (an anticoagulant, sometimes known as 'blood thinners'), and reduces the risk of stroke. About 10% of strokes occur in people unaware that they have AF."

For peer review only

What do you think having AF might mean for someone?
Prompts:
- *What it might be like to have an irregular heart beat*
- *Fears or concerns about living with AF*

What would you think about taking regular 'blood thinning' medication for AF?
Prompts:
- *An example medication is warfarin – what do you think about this medication?*

What do you think about the link AF has with stroke?
Prompts:
- *What experiences do people have with stroke?*
- *What are people's reactions to or concerns about stroke?*
- *What might it be like living with AF, knowing it increases the risk of stroke?*

C. Any other areas of discussion

Do you have any other areas, which we have not yet covered, which you would like to discuss in relation to this topic?

Interview close

- Thank participants for taking part in the study and remind them that their responses will remain confidential
- Ask participants if they have any questions.
- Let participants know they can contact the research team at any time using the details on the information leaflet, including if /d they have any further questions or wish to remove their responses from the study.

For peer review only

Interview 2 (post screening invitation)

A. Screening for atrial fibrillation

As you know, this interview is part of a research study we are undertaking to explore the offer of screening for AF in primary care. What were your first impressions on receiving the invitation to be screened for AF?

Prompts

- *Did you know anything about AF before you received the screening invite?*
- *Did you feel there was sufficient information in the screening invite?*

Do you think there might be any benefits of screening for AF?

Prompts:

- *Potential treatment of AF*
- *Stroke prevention*

Do you think there might be any harms or negative consequences of screening for AF?

Prompts:

- *Potential harms: psychological, false-positives, overdiagnosis, overtreatment, reduced quality of life, financial, opportunity costs*

Do you think this screening is relevant to you?

Prompt:

- *If so, why?*
- *If not, why not?*

Are you planning to go along for screening?

Prompt:

- *If so, why?*
- *If not, why not?*

Would you change anything about your life if screening showed you did have AF?

Prompt:

- *Lifestyle changes (routine/exercise/diet/smoking)*

B. Any other areas of discussion

Do you have any other areas, which we have not yet covered, which you would like to discuss in relation to this topic?

For peer review only

Interview 3 (Post screening)

A. Experiences of screening for AF

I'd like to explore with you your experiences of the AF screening programme. What were your first impressions on receiving the invitation to be screened for AF?

Prompts

- *Did you know anything about AF before you received the screening invite?*
- *Did you feel there was sufficient information in the screening invite?*
- *Did you feel the screening was relevant to you?*
- *If declined, why was this? What were your concerns about the process?*

Now I'd like to talk a little bit about what happened during the screening process [to be asked of screening attenders]

Prompts

- *What prompted you to go along?*
- *Did you understand what screening might involve?*
- *Did the doctor/nurse explain it all clearly to you, do you think?*
- *How did you find using the Zenicor device?*
- *Did you have any particular difficulties or challenges taking the readings you were asked to complete?*
- *Were you concerned about anything during the screening process? What was this in particular? Did you let anyone know about your concerns? Were these concerns addressed?*
- *What was good about your overall experience? Was there anything that was not so good about your experience?*
- *Did you understand how you were going to be informed about the outcome of your screening? If not, why not? How could things have been done differently?*
- *How did taking part in the screening make you feel?*

Given your experiences, do you think there are any benefits of screening for AF?

Prompts:

- *Potential treatment of AF*
- *Stroke prevention*

Do you think there might be any harms or negative consequences of screening for AF?

Prompts:

- *Potential harms: psychological, false-positives, overdiagnosis, overtreatment, reduced quality of life, financial, opportunity costs*

Would you recommend this screening programme to a find or family member?

Prompts:

- *If so, why?*
- *If not, why not?*

C. Any other areas of discussion

Do you have any other areas, which we have not yet covered, which you would like to discuss in relation to this topic?

For peer review only

COREQ (COnsolidated criteria for REporting Qualitative research) Checklist

A checklist of items that should be included in reports of qualitative research. You must report the page number in your manuscript where you consider each of the items listed in this checklist. If you have not included this information, either revise your manuscript accordingly before submitting or note N/A.

Topic	Item No.	Guide Questions/Description	Reported on Page No.
**Domain 1: Research team and reflexivity**			
Personal characteristics			
Interviewer/facilitator	1	Which author/s conducted the interview or focus group?	
Credentials	2	What were the researcher's credentials? E.g. PhD, MD	
Occupation	3	What was their occupation at the time of the study?	
Gender	4	Was the researcher male or female?	
Experience and training	5	What experience or training did the researcher have?	
Relationship with participants			
Relationship established	6	Was a relationship established prior to study commencement?	
Participant knowledge of the interviewer	7	What did the participants know about the researcher? e.g. personal goals, reasons for doing the research	
Interviewer characteristics	8	What characteristics were reported about the inter viewer/facilitator? e.g. Bias, assumptions, reasons and interests in the research topic	
**Domain 2: Study design**			
Theoretical framework			
Methodological orientation and Theory	9	What methodological orientation was stated to underpin the study? e.g. grounded theory, discourse analysis, ethnography, phenomenology, content analysis	
Participant selection			
Sampling	10	How were participants selected? e.g. purposive, convenience, consecutive, snowball	
Method of approach	11	How were participants approached? e.g. face-to-face, telephone, mail, email	
Sample size	12	How many participants were in the study?	
Non-participation	13	How many people refused to participate or dropped out? Reasons?	
Setting			
Setting of data collection	14	Where was the data collected? e.g. home, clinic, workplace	
Presence of non-participants	15	Was anyone else present besides the participants and researchers?	
Description of sample	16	What are the important characteristics of the sample? e.g. demographic data, date	
Data collection			
Interview guide	17	Were questions, prompts, guides provided by the authors? Was it pilot tested?	
Repeat interviews	18	Were repeat inter views carried out? If yes, how many?	
Audio/visual recording	19	Did the research use audio or visual recording to collect the data?	
Field notes	20	Were field notes made during and/or after the inter view or focus group?	
Duration	21	What was the duration of the inter views or focus group?	
Data saturation	22	Was data saturation discussed?	
Transcripts returned	23	Were transcripts returned to participants for comment and/or	

For peer review only

Topic	Item No.	Guide Questions/Description	Reported on Page No.
		correction?	
**Domain 3: analysis and findings**			
Data analysis			
Number of data coders	24	How many data coders coded the data?	
Description of the coding tree	25	Did authors provide a description of the coding tree?	
Derivation of themes	26	Were themes identified in advance or derived from the data?	
Software	27	What software, if applicable, was used to manage the data?	
Participant checking	28	Did participants provide feedback on the findings?	
Reporting			
Quotations presented	29	Were participant quotations presented to illustrate the themes/findings? Was each quotation identified? e.g. participant number	
Data and findings consistent	30	Was there consistency between the data presented and the findings?	
Clarity of major themes	31	Were major themes clearly presented in the findings?	
Clarity of minor themes	32	Is there a description of diverse cases or discussion of minor themes?	

Developed from: Tong A, Sainsbury P, Craig J. Consolidated criteria for reporting qualitative research (COREQ): a 32-item checklist for interviews and focus groups. *International Journal for Quality in Health Care*. 2007. Volume 19, Number 6: pp. 349 – 357

Once you have completed this checklist, please save a copy and upload it as part of your submission. DO NOT include this checklist as part of the main manuscript document. It must be uploaded as a separate file.

For peer review only

Why do people take part in atrial fibrillation screening? Qualitative interview study in English primary care

Journal:	*BMJ Open*
Manuscript ID	bmjopen-2021-051703.R2
Article Type:	Original research
Date Submitted by the Author:	08-Feb-2022
Complete List of Authors:	Hoare, Sarah; The Healthcare Improvement Studies Institute, Department of Public Health and Primary Care, University of Cambridge<br>Powell, Alison; The Healthcare Improvement Studies Institute, Department of Public Health and Primary Care, University of Cambridge<br>Modi, Rakesh; University of Cambridge Primary Care Unit, Department of Public Health and Primary Care, University of Cambridge<br>Armstrong, Natalie; University of Leicester, Department of Health Sciences<br>Griffin, Simon; University of Cambridge Primary Care Unit, Department of Public Health and Primary Care, University of Cambridge<br>Mant, Jonathan; University of Cambridge Primary Care Unit, Department of Public Health and Primary Care, University of Cambridge<br>Burt, Jenni; The Healthcare Improvement Studies Institute, Department of Public Health and Primary Care, University of Cambridge<br>SAFER Authorship Group, The; SAFER Programme
**Primary Subject Heading**:	Public health
Secondary Subject Heading:	General practice / Family practice, Cardiovascular medicine, Public health, Health services research
Keywords:	PRIMARY CARE, CARDIOLOGY, PUBLIC HEALTH, QUALITATIVE RESEARCH

SCHOLARONE™
Manuscripts

I, the Submitting Author has the right to grant and does grant on behalf of all authors of the Work (as defined in the below author licence), an exclusive licence and/or a non-exclusive licence for contributions from authors who are: i) UK Crown employees; ii) where BMJ has agreed a CC-BY licence shall apply, and/or iii) in accordance with the terms applicable for US Federal Government officers or employees acting as part of their official duties; on a worldwide, perpetual, irrevocable, royalty-free basis to BMJ Publishing Group Ltd ("BMJ") its licensees and where the relevant Journal is co-owned by BMJ to the co-owners of the Journal, to publish the Work in this journal and any other BMJ products and to exploit all rights, as set out in our licence.

The Submitting Author accepts and understands that any supply made under these terms is made by BMJ to the Submitting Author unless you are acting as an employee on behalf of your employer or a postgraduate student of an affiliated institution which is paying any applicable article publishing charge ("APC") for Open Access articles. Where the Submitting Author wishes to make the Work available on an Open Access basis (and intends to pay the relevant APC), the terms of reuse of such Open Access shall be governed by a Creative Commons licence – details of these licences and which Creative Commons licence will apply to this Work are set out in our licence referred to above.

Other than as permitted in any relevant BMJ Author's Self Archiving Policies, I confirm this Work has not been accepted for publication elsewhere, is not being considered for publication elsewhere and does not duplicate material already published. I confirm all authors consent to publication of this Work and authorise the granting of this licence.

For peer review only

Why do people take part in atrial fibrillation screening? Qualitative interview study in English primary care

Sarah Hoare[1] (BA, MPhil, PhD. Research Associate. https://orcid.org/0000-0002-8933-217X)

Alison Powell[1] (MA, PhD. Research Associate. https://orcid.org/0000-0003-2524-5357)

Rakesh Modi[2] (MBBS MA MRCP MRCGP DCH DRCOG MSc. Academic GP and Wellcome Trust PhD Fellow. ORCID: https://orcid.org/0000-0001-9651-6690)

Natalie Armstrong[3] (BA, MSc, PhD. Professor of Healthcare Improvement Research. https://orcid.org/0000-0003-4046-0119)

Simon Griffin[2] (MBBS MSc DM FRCGP FMedSci Professor of General Practice. http://orcid.org/0000-0002-2157-4797)

Jonathan Mant[2] (MD, FFPH. Professor of Primary Care Research. https://orcid.org/0000-0002-9531-0268)

Jenni Burt[1] (PhD. Senior Social Scientist. https://orcid.org/0000-0002-0037-274X)

and The SAFER Authorship Group.

[1]THIS Institute (The Healthcare Improvement Studies Institute), University of Cambridge, Cambridge Biomedical Campus, Clifford Allbutt Building, Cambridge CB2 0AH, UK

[2]Primary Care Unit, Department of Public Health & Primary Care, University of Cambridge School of Clinical Medicine, Strangeways Research Laboratory, University of Cambridge, Worts Causeway, Cambridge CB1 8RN, UK

[3]SAPPHIRE Research Group, Department of Health Sciences, University of Leicester, George Davies Centre, University Road, Leicester LE1 7RH, UK

Correspondence to: Sarah Hoare, THIS Institute, University of Cambridge, Cambridge Biomedical Campus, Clifford Allbutt Building, Cambridge CB2 0AH, UK. Sarah.hoare@thisinstitute.cam.ac.uk

Word count: abstract: 299; manuscript: 4168

Number of references: 74

Number of figures: 1 **Number of tables:** 1

For peer review only

Statements

Acknowledgements

The SAFER Authorship Group includes:

Andrew Dymond, University of Cambridge, UK

Richard Hobbs, University of Oxford, UK

Rachel Johnson, University of Bristol, UK

Richard McManus, University of Oxford, UK

Kate Williams, University of Cambridge, UK

We thank the SAFER participants for their time and for sharing their experiences of taking part in screening. We also thank the GP practices involved and the local National Institute for Health Research Clinical Research Network for supporting this study. We are grateful to the SAFER study team and in particular to Millie Watson for her work in administering these interviews, and to James Brimicombe for managing the electronic SAFER data.

Ethical approval

The study was approved by the London-Central NHS Research Ethics Committee (reference number: 18/LO/2066).

For peer review only

Why do people take part in atrial fibrillation screening? Qualitative interview study in English primary care

For peer review only

Abstract

Objectives: There is insufficient evidence to support national screening programmes for atrial fibrillation (AF). Nevertheless, some practitioners, policymakers and special interest groups have encouraged introduction of opportunistic screening in primary care in order to reduce the incidence of stroke through earlier detection and treatment of AF. The attitudes of the public towards AF screening are unknown. We aimed to explore why AF screening participants took part in the screening.

Design: Semi-structured longitudinal interview study of participant engagement in the SAFER study (Screening for Atrial Fibrillation with ECG to Reduce stroke). We undertook initial interviews face-to-face, with up to two follow-up telephone interviews during the screening process. We thematically analysed and synthesised this data to understand shared views of screening participation.

Setting: 5 primary care practices in the East of England, UK.

Participants: 23 people taking part in the SAFER study first feasibility phase.

Results: Participants were supportive of screening for AF, explaining their participation in screening as a ‘good thing to do’. Participants suggested screening could facilitate earlier diagnosis, more effective treatment, and a better future outcome, despite most being unfamiliar with AF. Participating in AF screening helped attenuate participants’ concerns about stroke and demonstrated their commitment to self-care and being a ‘good patient’. Participants felt that the screening test was non-invasive, and they were unlikely to have AF; they therefore considered engaging in AF screening was low risk, with few perceived harms.

Conclusions: Participants assessed the SAFER AF screening programme to be a legitimate, relevant, and safe screening opportunity, and complied obediently with what they perceived to be a recommendation to take part. Their unreserved acceptance of screening benefit and lack of awareness of potential harms suggests that uptake would be high but reinforces the importance of ensuring participants receive balanced information about AF screening initiatives.

Keywords: screening, qualitative research, atrial fibrillation

Strengths and Limitations:

- Our research adds to the limited evidence base about atrial fibrillation (AF) screening participation: opportunistic AF screening is encouraged in primary care to reduce the incidence of stroke through earlier detection and treatment of AF, despite insufficient evidence currently available to support systematic AF screening.
- We report the views of people taking part in AF screening as part of a research study, limiting the relevance of our findings for understanding public engagement in either opportunistic AF screening conducted as part of routine primary care or potential future systematic AF screening programmes.
- This was a study of people who participated in AF screening and it does not address the views of those who were invited and opted to not take part.
- The lack of ethnic diversity in our sample reduces the utility of the results, particularly because attitudes towards screening and AF are known to differ by ethnic background.
- Our research contributes to social science literature about the public's 'moral obligation' to participate in screening even when both the programme and the condition are largely unfamiliar to participants.

For peer review only

I. Introduction

Opinion on whether there should be a screening programme for Atrial Fibrillation (AF) is divided [1–3]. Screening advocates include clinical societies [4–7], patient associations [8], clinicians [9,10], and pharmaceutical and technology companies [11,12]. Justifications for AF screening are well-rehearsed. Having AF (a common, often asymptomatic, heart arrhythmia) increases the risk of stroke five-fold [13] with AF-related stroke typically more severe than non-AF-related stroke [14]. AF anticoagulant treatment is effective at reducing stroke risk [15], and screening devices for use in the community are inexpensive [2]. Conversely, caution around AF screening is attributed to insufficient evidence on effectiveness, cost-effectiveness and potential harms of national systematic AF screening programmes, which are not currently recommended [16,17]. Questions remain, for example, about the difference in stroke risk and anticoagulation benefit for people with AF detected by screening rather than in routine care [16,18].

In the meantime, opportunistic AF screening is encouraged in primary care, leading to accusations of 'back-door' screening [12]. Opportunistic screening occurs through many routes. In the UK, clinicians are recommended to assess patients' pulse rhythm as part of NHS health checks [19], driven by an explicit aim of NHS programmes and policy to increase identification and diagnosis of previously undetected AF [20,21]. NHS England has distributed digital ECG devices to increase AF detection [22], and some clinical commissioning groups have encouraged opportunistic AF case-finding in primary care [23,24].

The public's views have largely been absent from this debate. Patient associations which champion AF screening provide important information from the perspective of a motivated minority, but tell us little about the opinions of the wider public [12]. If typically positive attitudes to screening programmes apply here [25,26], it seems likely that the public will be enthusiastic about AF screening, as seen in a small-scale study of AF screening trial participants [27] and informal reports from a previous AF screening trial [28]. However, little is known about the reasons why the public are motivated to participate in AF screening [27,28]. We aimed to explore why participants in SAFER, an AF screening study, opted to take part.

II. Methods

A. Design and participants

Our data are from a longitudinal interview study with participants in the AF screening study SAFER (Screening for Atrial Fibrillation using ECG to Reduce stroke) (https://www.safer.phpc.cam.ac.uk/). We interviewed participants during their study involvement to explore their experience of AF screening, including their views on why they had taken part.

The SAFER programme is ongoing, and interviews were conducted as part of a feasibility study. Participants were drawn from five participating GP (General Practitioner) practices in the East of England; eligible patients were aged 65 years and over, not taking anticoagulant medication, and were neither on a palliative care register nor living in a residential (care) home. Participants were first invited to contribute to SAFER research and subsequently to take part in AF screening. The screening involved using a Zenicor (www.zenicor.com) hand-

For peer review only

held single-lead electrocardiogram (ECG) device four times a day for between one and four weeks.

We selected interview participants from the first wave of feasibility phase participants. Potential interviewees were purposively sampled by age, gender and GP practice to ensure a varied sample. All participants were invited by letter and agreed to take part in SAFER and, on invitation to screening, elected to take part.

B. Data collection

We interviewed participants up to three times throughout their screening. Interview one occurred shortly after participants agreed to take part in the SAFER research study; interview two after a participant had received their invitation to take part in SAFER screening; and interview three after they had completed the screening. Interview one was held either at the participant’s home or at their GP practice, and interviews two and three were conducted by telephone. Not all participants took part in all interviews: participants could elect not to take part in each interview phase, and some participants were not invited to the second interview to ensure that they had the opportunity to participate in screening (see Figure 1). Interviews were typically held with only the interviewer ([author 1]) and interview participant, but when participants’ partners were present they were included in the discussion and consented to take part. Fieldnotes were completed following each interview.

Interviews were semi-structured and used a flexible topic guide exploring experiences of, and attitudes towards, screening in general and AF screening in particular (see supplemental material). These topics were explored by participants throughout all three interviews. The topic guide was designed to reflect our research question and was refined and iteratively adapted as interviews progressed to accommodate areas of interest raised by participants. Consent was taken prior to each interview. Interviews were conducted in 2019 and continued until we had reached sufficient information power [29] to have a meaningful dataset. Interviews lasted on average 30 minutes each (range 10-90 minutes) and were audio-recorded. We completed 53 interviews with 23 participants (interview 1 n=23, interview 2 n=10, interview 3 n=20). Table 1 lists participants’ characteristics.

[Figure 1: Flowchart of interviews in the screening process]

[Table 1: Socio-demographics of participants]

C. Analysis

Interviews were transcribed verbatim and analysed using a thematic approach to explore the reasons why people took part in AF screening. Interviews were collated, and the initial analysis phase focused on 16 transcripts selected for their relevance to addressing the question and drawn from all three interview time-points (interview 1, 7 transcripts; interview 2, 3 transcripts; interview 3, 6 transcripts, with some participants represented more than once). This initial analysis was followed by analysis of the whole interview dataset (n=53). SH conducted coding, on paper and then supported by the software NVivo 12, with codes generated inductively from topics raised by participants and interview fieldnotes, and deductively from the interview schedule. We developed key themes through consensus meetings between SH, AP and JB, and SH then explored these themes within the wider dataset to establish the veracity of key themes and identify deviant cases, with the themes subsequently refined. These themes were synthesised to understand shared views of screening participation, aided by reference to social science and health screening literature about participation in screening [30–38].

D. Patient and public involvement (PPI)

The SAFER programme is guided by four PPI representatives [39] who have been involved since the programme was conceived and funded. The representatives are embedded within the team (e.g. one is a co-applicant, another sits on the programme steering committee) and advise on all aspects of the design, management and delivery of the programme, including interpreting and disseminating the findings. We have also established a supplementary qualitative work-stream PPI group with whom we regularly consult about research design questions.

III. Results

Quotations in the text are followed by the participant's interview ID number (1-53), practice code (A-E) and interview phase (1-3).

A. Summary

Engagement with AF screening within the SAFER study was driven by several inter-connected considerations, many relating to wider attitudes that screening is a 'good thing' to participate in, and to the perceived importance of preventive health behaviours. Participants knew little about AF, but were motivated to be screened by the perceived potential for reducing their risk of catastrophic disability associated with stroke, and a seemingly low-risk screening test with few if any apparent harms and significant perceived benefits.

B. The importance of screening in general

Participants were familiar with national screening programmes, often referring to their own or a relative's prior experience of taking part in screening. Screening was described as a "no-brainer" by several participants, and it was clear within our sample that screening was seen as a routine healthcare activity in which participation was the only reasonable option. This option was driven by a desire to follow clinical recommendations, gain insight into health status and, if required, access early treatment, and was further underpinned by a 'fear of missing out' by not participating.

1. Clinically recommended screening

Screening invitations were typically seen as legitimate requests from a clinical authority which was presumed to recommend the screening programme. Participants explained that this meant that screening was something they ought to at least consider engaging with, and for a minority as a mandate that they must comply with.

> *"And I assume when something like that [a screening invitation] comes from the post, I immediately comply, I don't question. I don't question because it's come from a…you know, it's a bit different to somebody trying to ring me up and telling me that they can spend my pension more successfully than I can. It comes from an authoritative source, and I don't think there's any question of me not believing what the prospects are." (05B_1)*

2. Reassurance of screening

Participants expected screening would provide them with reassurance about their health. This could be definitive, with a negative test result providing "peace of mind" that the screened for condition was "something else you can tick off the list of things to worry about" (06E_1). Reassurance could also be more anticipatory, whereby participation in screening was protective of regret if in the future one did indeed get diagnosed with the condition but

For peer review only - http://bmjopen.bmj.com/site/about/guidelines.xhtml

had chosen not to be screened. Simply attending screening also seemed to confer reassurance about healthiness, whereby participants additionally acknowledged responsibility for maintaining their health and preventing ill-health:

> *"But, I think, it's a brilliant idea and the thing is though, that things can happen in between, so you've still got to be responsible for your own health."*
> *(25A_1)*

3. Early diagnosis, early treatment, better outcomes

Participants were clear that early identification of a screened condition would enable preventive action or prompt treatment to avert ill-health or reduce disease severity. Participants placed different emphases on the importance of these potentialities (from prevention to treatment) but were united by a presumption that earliness was advantageous. By comparison, 'late' identification or treatment was associated with perceived poorer outcomes for individuals and the healthcare system. Participants often drew on this binary conceptualisation of early versus late identification to explain why it was sensible to take part in screening:

> *"I'd rather find something out, if there is a screening thing that can indicate early symptoms that can be caught early and something can be done about it, I think I'd much rather have that option than sort of find out later, you know, further down the road that, well it's too late now." (16D_1)*

a) Identifying silent conditions: robust test

Underlying perceived benefits of early diagnosis was participants' awareness that screening could identify a condition before symptoms could be noticed or manifested, or for which symptoms were absent (a 'silent' condition):

> *"I get this thing for the bowel screening, which isn't as much fun. And despite that, I've gone in for it, because as a layman, I simply have no idea what's going on inside my body. The fact I feel fit, doesn't mean I am fit, you know, from those sort of complaints. [I: So…] So the answer is, I've said yes to that [bowel cancer screening] and I would have said yes to this [AF screening]."*
> *(11A_2)*

The ability of screening to identify silent conditions led to a view amongst some participants that screening tests could provide a particularly in-depth or robust review of health. For a few this extended the scope beyond the screened condition; here, the SAFER AF screening came to be viewed as a "free medical" (15A_3). Screening thus offered ready access to healthcare and perceived "health checks" without the need to present tangible health symptoms:

> *R: "I think, I don't want to bother the doctor he's so busy, so I won't bother to go until I really think, I'm going to have to go. […] I think, a lot of people don't go, so screening, perhaps, would ensure that they are seen for a particular thing, once every three years or however they're done."*
>
> *I: "And, do you think going to a screening appointment is different to just going for something else?"*

R: "Well, I think, it has to be 'cause I don't think anybody, unless they've got any symptoms, has any reason to think they've got anything."
(25A_1)

b) Not taking part

Associated with reducing the risk of 'late' diagnosis, participating in screening also protected participants from moral judgement about *not* engaging in screening. Non-screeners were variously portrayed by participants as unduly embarrassed, uninformed, indolent, irresponsible, wilfully ignorant, or gratuitously anxious, and often a combination of these. A significant part of participants' moralising about non-screeners was concern that they were missing out on the benefit of screening:

"I mean maybe there are some people that don't want to know. They live in a dream world and…but I just think if you've got the chance to do something about it and it gives you a better quality, even if it's only a short one, that's great, you should grab it."
(15A_3)

C. AF screening

1. Views about AF

AF was a new condition to most participants, except those with first-hand experience as a result of a family member's diagnosis or prior clinical knowledge. Some participants struggled to pronounce the term and many explained that they had conducted internet research to better understand the condition, supplementing the study literature provided. Despite this, after interviewer explanation, participants engaged with the concept of an irregular heartbeat and described what they thought it might be like to have the condition. At best, participants understood AF as a benign condition, but most expressed concern about the association with stroke. Participants anticipated that, if they had AF, they would worry about having a major and sudden heart-related incident, and expected having to modify their lifestyle (for some to a very significant and limiting degree) and to receive corrective treatment to address it:

I: […] "I wonder what you thought it might be like to have an irregular heartbeat?"

R: "A little bit disconcerting. I would say you would want it regulated, maybe if it's to have a pacemaker fitted or something. You wouldn't want to live with something that you know…you'd probably be able to feel it beating faster. You wouldn't want to think to yourself, am I having a stroke shortly? So I would say it's more peace of mind to know that you can regulate it if you have a… I mean, if you know that your heart is beating faster and it could be a start of a stroke, and you can take a pill to stop it and you didn't know about it, you'd be a bit miffed to say if you didn't have one."
(06E_1)

2. Views about AF screening

Most participants did not expect to receive a diagnosis of AF as a result of taking up the offer of screening. They explained this in relation to their own perception of their health, whether because they were healthy overall, or because they identified an absence of AF symptoms or prior heart-related issues. Despite this, participants explained that participating in screening was useful because there was a *chance* that they may unknowingly have AF. As a

For peer review only - http://bmjopen.bmj.com/site/about/guidelines.xhtml

silent condition with perceived serious consequences and a presumption for curative treatment, participants recognised the utility of checking to see if they had it:

> *I: "Do you think the atrial fibrillation screening is relevant to you?"*
>
> *R: "I don't suffer from any of those symptoms. That's as far as I can…or I'm not aware of suffering from those symptoms. That's as far as I can go. Like a lot of things that are more evident with age, I think it ill behoves me to say, oh, I'm, you know, I don't need to be tested because I feel fine." (11A_2)*

3. Stroke fear

Participants were clear that stroke was not a condition they wished to experience. The consequences of stroke were recognised to be potentially debilitating and life-changing. Whilst those who knew people who had had a stroke often recognised a diversity of recovery experiences that included more positive outcomes, there remained a concern about the high level of disability stroke could cause.

Participants typically recognised both that a stroke could happen unexpectedly "to anybody at any time, any day, any age really" (16D_1) and that there were genetic and modifiable lifestyle factors which could increase the risk of having a stroke. Together with concern about the consequences of having a stroke, this dual conception of stroke as preventable and unexpected made the association between AF and stroke significant, and thus AF screening particularly worthwhile:

> *I: "What do you think about the link between atrial fibrillation and stroke?"*
>
> *R: "I would try to do everything I can not to have a stroke. So if there was something I could do that would stop me turning into a vegetable, I will do it. So as far as I'm concerned, it's a no-brainer. Once you've had the stroke, whatever life you have got left, it's not going to be much fun, so try and avoid it." (06E_1)*

4. Low-risk screening

The AF screening test involved participants placing their thumbs or two fingers on a portable ECG device. Participants unanimously recognised this to be non-invasive and typically understood the screening to be safe and to not cause harm, often aligning it with the ease of measuring blood pressure.

Participants acknowledged that anxiety around the screening test may arise for others. However, they frequently related this to the screened individuals' psychological state rather than something inherent to the testing process. Participants rarely recognised other iatrogenic screening harms. If they did, such harms were presented as legitimate concerns and often as intrinsic to testing. However, participants presumed the screening was safe and, when discussed, positioned consideration of these issues as the responsibility of the screening provider.

Compared to the perceived fuzzy and inconsequential harms of screening, participants saw the advantages of AF screening as tangible and significant. Screening could identify AF 'early', using a test that was perceived to cause no harm, and which could ameliorate the risk of stroke:

> *R1: "Any harm? No I don't think that any harm can arise from that all, personally, screening, no."*

For peer review only

> *R2: "I mean to say, it looks as if just by putting your thumbs on the actual screening test on the box, you're not going to get anything invasive from that. And, I say, if it shows up something that can be dealt with sooner rather than later, that can only be a good thing in our view really." (16D_2)*

IV. Discussion

A. Summary

We explored the reasons why people took part in AF screening through interviews with SAFER study participants. Their accounts presented screening as a routine obligation and something one 'ought' to do as a responsible to ensure good health and prevent illness. Limited awareness of AF did not detract from their view of the utility of AF screening – rather, preventing stroke was a strong rationale for participating. Participants assessed AF screening to be a legitimate, relevant and safe screening opportunity, and complied obediently with what they perceived to be a recommendation to take part.

B. Strengths and limitations

Our research adds to the limited evidence about AF screening participation. Our findings also contribute to social science literature about patients' 'moral obligation' [30] to participate in screening [30–33], even when both the programme and the condition are largely unfamiliar to participants.

It is likely that our results reflect the delivery of SAFER as a research study. Whilst the SAFER study has been designed to mimic a national screening programme, participants were aware that it was research. This may limit the relevance of our findings for understanding public engagement in either opportunistic AF screening conducted as part of routine primary care, or potential future systematic AF screening programmes. For example, participants reported that a key reason for taking part was to 'help out', a prevalent and well-recognised motivation for participating in research [40].

This was a study of people who participated in AF screening and does not address the views of those who were invited and opted to not take part. Nevertheless, focusing on people who did engage has allowed us to understand the information needs and potential misconceptions of this population.

Participants' familiarity with, and participation in, national screening programmes suggests their enthusiasm for AF screening will not be relevant to all screening invitees. For example, recent studies in the UK found that prior attendance in screening is positively correlated with future participation.[41,42] Participants in our study match national trends by being less deprived by practice deprivation status [43]. The lack of ethnic diversity in our sample will also necessarily reduce the utility of the results, particularly because screening uptake and attitudes towards AF differ by ethnic background [43,44].

C. Comparison with existing literature

Participants' concern about having a stroke mediated their unfamiliarity with AF and encouraged them to take part. The novelty of AF is unsurprising as studies find limited awareness of the condition, whether the participants' relationship to AF is: just at risk [45]; being screened for it [27]; prior to diagnosis [46]; or having it [44,47]. Similarly, those with AF report being worried or fearful about their condition [46,47], consider it a serious heart

For peer review only

disease [48] and overestimate the risk of stroke [49]. AF was explained to participants in the context of stroke-risk, and this may explain participants' worries about AF-induced stroke: the majority of AF patients in a 2002 study [44] did not consider their condition to be severe, but almost half were not aware that having AF predisposed them to stroke.

The enthusiasm participants had for AF screening, and the weighting they attributed to the benefits versus the harms of screening, accord with other studies on the public's experience of national screening programmes [50,51]. There is widespread public support for screening even in survey scenarios in which there was no treatment for the screened condition [25,26], or if screening is not clinically recommended because participants are outside screening age thresholds [52,53]. The benefits SAFER participants attributed to AF screening concur with reported experience. Screening offers the hope of reassurance [30,34,35] and confirmation of healthiness [34], or in the worst case, the benefit in knowing one's ill-health status [54] and associated advantageous early diagnosis or treatment [55]. Participation also offers an 'insurance policy' [56], whereby the often presumed 'health check' [35] means those taking part can be assured that 'they had done everything they could' [34] if they later develop the screened condition. Like SAFER participants, this enthusiasm is contextualised by reported limited concern or awareness of screening harms [55]. A recent US study found that many respondents could not name any harms of screening, and where they could, focused, as SAFER participants and other AF screening participants did [27], on the direct harms of the screening test itself [57]. Even when harms are recognised, these are typically of a lower priority to participants compared to the benefits of screening [58].

Informing participants' consideration of the harms and benefits of screening was an assumption that taking part in screening was something they ought to do, corresponding with evidence from screening decision-making studies [53,59–62]. Sociological work has shown how screening participation is associated with maintaining, and being responsible for, one's own health [30–38], and part of patients' efforts to be a 'good patient' and to use healthcare resources appropriately [63].

D. Implications for practice and research

The public can be expected to be unfamiliar with AF, to anticipate that screening is recommended, to perceive any AF test as a comprehensive heart function review and to be positive about screening. Clinicians should be prepared to work with members of the public interested in screening to help them understand what they are being offered and what the risks and benefits of taking part are. SAFER participants suggested that early identification leads to better results. Counselling individuals on the benefits and risks of AF screening is particularly pertinent given participant expectations for curative treatment and because clinical evidence on the benefit of managing screen-detected AF is still uncertain [18]. As with all screening, benefits must be weighed against harms [64].

Receiving a positive result may be unexpected for those engaging in screening for health confirmation, and could engender feelings of health vulnerability [65]. These participants may need help to moderate concerns about the severity and lifestyle impact of the condition. Clinician engagement is significant because their reactions and approaches to AF have been found to impact patients' perceptions of their condition [66], and patients' understanding of AF may be aided by often called-for patient education interventions [44,49,67,68]. Negative test results may confer 'healthiness' [69] and induce false reassurance about their stroke risk. Though the evidence-base is small [70] it is plausible that participants' existing 'unhealthy' behaviours are validated as acceptable and continued [71], whilst any future AF-related symptoms may be 'downplayed' [72], discouraging prompt healthcare seeking. Consequently, clinicians are recommended to contextualise negative results with reference to the necessary limitations of any screening test and participants' ongoing risk of developing the condition [69].

For peer review only - http://bmjopen.bmj.com/site/about/guidelines.xhtml

Our study has contributed to a small evidence-base on public experience of AF screening [27]. If AF screening is demonstrated to be effective, then complementary studies would be important to understand the attitudes of people who do not participate in screening and how participants (particularly those with positive results) weigh up the benefits and harms throughout the screening process.

For peer review only

V. Tables

Table 1: Socio-demographics of participants (n=23)

Participant characteristic	*n*
**Age group**	
65-69 years	9
70-74 years	7
75-79 years	5
80+ years	2
**Sex**	
Female	10
Male	13
**Practice** (*Deprivation score 1-10; 10 = least deprived)*[1]	
A[2] *(10)*	8
B *(8)*	5
C *(8)*	4
D *(6)*	4
E *(5)*	2

[1] The deprivation score is taken from National General Practice Profiles [73], using the English Indices of Deprivation to calculate the Index of Multiple Deprivation, which provides "an overall measure of deprivation experienced by people living in an area" [73,74]
[2] Practice name pseudonym

For peer review only

VI. Contributions

JB planned the interview study and oversaw the data collection by SH. SH led the analysis with themes developed with JB and AP, and contributions from RM. SH and JB drafted the original manuscript. SH, AP, RM, NA, SG, JM, JB critically reviewed, revised, and approved the final version. All members of the SAFER Authorship Group (AD, RH, RJ, RM, KW) reviewed and approved the initial submission and had the opportunity to comment, and the final version was shared with them.

VII. Competing interests

JM has received honoraria from BMS/Pfizer.

VIII. Funding

The SAFER study is funded by the National Institute for Health Research (NIHR) Programme Grants for Applied Research (grant reference number RP-PG-0217-20007) and School for Primary Care Research (SPCR-2014-10043, project 410). The views expressed are those of the authors and not necessarily those of the NIHR or the Department of Health and Social Care.

RM is supported by the Wellcome Trust as part of the Wellcome Trust PhD Programme for Primary Care Clinicians (grant reference number 203921/Z/16/Z).

NA is supported by a Health Foundation Improvement Science Fellowship (N/A award/grant number) and also by the National Institute for Health Research (NIHR) Applied Research Collaboration East Midlands (ARC EM) (N/A award/grant number). The views expressed are those of the authors and not necessarily those of the NHS, the NIHR or the Department of Health and Social Care.

The University of Cambridge has received salary support in respect of SG from the NHS in the East of England through the Clinical Academic Reserve (N/A award/grant number).

JM is an NIHR Senior Investigator (N/A award/grant number).

SH, AP and JB are based in The Healthcare Improvement Studies Institute (THIS Institute), University of Cambridge. THIS Institute is supported by the Health Foundation, an independent charity committed to bringing about better health and healthcare for people in the UK (N/A award/grant number).

For peer review only

Data sharing

The data may be available on request from the authors, subject to appropriate permissions: please email the PI Jenni Burt (jenni.burt@thisinstitute.cam.ac.uk) for details.

IX. References

1. Neubeck L, Orchard J, Lowres N, Freedman SB. To Screen or Not to Screen? Examining the Arguments Against Screening for Atrial Fibrillation. Heart Lung Circ. 2017 Sep;26(9):880–6.

2. Lown M, Garrard J, Irving G, Edwards D, Hobbs FR, Mant J. Should we screen for atrial fibrillation? British Journal of General Practice. 2017 Jul;67(660):296–7.

3. Lown M, Moran P. Should we screen for atrial fibrillation? BMJ. 2019 Feb 13;l43.

4. Hobbs FR, Taylor CJ, Jan Geersing G, Rutten FH, Brouwer JR. European Primary Care Cardiovascular Society (EPCCS) consensus guidance on stroke prevention in atrial fibrillation (SPAF) in primary care. Eur J Prev Cardiol. 2016 Mar;23(5):460–73.

5. Kirchhof P, Benussi S, Kotecha D, Ahlsson A, Atar D, Casadei B, et al. 2016 ESC Guidelines for the management of atrial fibrillation developed in collaboration with EACTS. Eur Heart J. 2016 Oct 7;37(38):2893–962.

6. Lip GY, Ramsay SG. Insights from the RCPE UK Consensus Conference on approaching the comprehensive management of atrial fibrillation. Expert Review of Cardiovascular Therapy. 2012 Jun 1;10(6):697–700.

7. Authors/Task Force Members, Camm AJ, Lip GYH, De Caterina R, Savelieva I, Atar D, et al. 2012 focused update of the ESC Guidelines for the management of atrial fibrillationAn update of the 2010 ESC Guidelines for the management of atrial fibrillation Developed with the special contribution of the European Heart Rhythm Association. Europace. 2012 Aug 24;14(10):1385–413.

8. Atrial Fibrillation Association, AntiCoagulation Europe. The AF Report. Atrial Fibrillation: Preventing A Stroke Crisis [Internet]. Atrial Fibrillation Association and AntiCoagulation Europe; 2011. Available from: http://atrialfibrillationassociation.org.uk/app/webroot/files/file/140508-cw-FINAL-The%20AF%20Report.pdf

9. Robson J, Schilling R. Atrial fibrillation: time for active case finding. British Journal of General Practice. 2019 Feb;69(679):58–9.

10. Kearney M, Fay M, Fitzmaurice DA. Stroke prevention in atrial fibrillation: we can do better. Br J Gen Pract. 2016 Feb 1;66(643):62–3.

11. Mahase E. Atrial fibrillation groups lobby patients to overturn screening committee decision. BMJ [Internet]. 2019 Jul 9 [cited 2020 Oct 2];366. Available from: https://www.bmj.com/content/366/bmj.l4612

12. McCartney M, Fell G, Finnikin S, Hunt H, McHugh M, Gray M. Why 'case finding' is bad science. J R Soc Med. 2019 Dec 12;014107681989142.

13. Wolf PA, Abbott RD, Kannel WB. Atrial fibrillation as an independent risk factor for stroke: the Framingham Study. Stroke. 1991 Aug;22:983–8.

For peer review only

14. Marini C, De Santis F, Sacco S, Russo T, Olivieri L, Totaro R, et al. Contribution of atrial fibrillation to incidence and outcome of ischemic stroke: results from a population-based study. Stroke. 2005 Jun;36:1115–9.

15. G. Hart R, A. Pearce L, I. Aguilar M. Meta-analysis: Antithrombotic Therapy to Prevent Stroke in Patients Who Have Nonvalvular Atrial Fibrillation. Annals of Internal Medicine [Internet]. 2007 Jun 19 [cited 2020 Nov 20]; Available from: https://www-acpjournals-org.ezp.lib.cam.ac.uk/doi/abs/10.7326/0003-4819-146-12-200706190-00007

16. King S, Fitzgerald A, Bartlett C, Mahon J, Arber M, Carr E, et al. Evidence Summary for Screening for Atrial Fibrillation in Adults: External review against programme appraisal criteria for the UK National Screening Committee. London: UK National Screening Committee; 2019.

17. US Preventive Services Task Force, Curry SJ, Krist AH, Owens DK, Barry MJ, Caughey AB, et al. Screening for Atrial Fibrillation With Electrocardiography: US Preventive Services Task Force Recommendation Statement. JAMA. 2018 Aug 7;320(5):478.

18. Jones NR, Taylor CJ, Hobbs FDR, Bowman L, Casadei B. Screening for atrial fibrillation: a call for evidence. Eur Heart J. 2020 Mar 7;41(10):1075–85.

19. NHS Health Check: Best practice guidance [Internet]. London: Public Health England; 2017 [cited 2020 Oct 2]. Available from: https://www.england.nhs.uk/london/wp-content/uploads/sites/8/2019/08/2.1.7-NHS-Health-Check-Best-Practice-Guidance-2017.pdf

20. NHS RightCare » High value intervention in atrial fibrillation [Internet]. [cited 2020 Oct 9]. Available from: https://www.england.nhs.uk/rightcare/products/pathways/cvd-pathway/af/

21. NHS. The NHS Long Term Plan [Internet]. 2019 [cited 2020 Sep 23]. Available from: https://www.longtermplan.nhs.uk/wp-content/uploads/2019/08/nhs-long-term-plan-version-1.2.pdf

22. Independent Evaluation of the AHSN Network mobile ECG rollout programme: Full Report. Southampton: Wessex Academic Health Science Network; 2019 p. 82.

23. Cole J, Torabi P, Dostal I, Homer K, Robson J. Opportunistic pulse checks in primary care to improve recognition of atrial fibrillation: a retrospective analysis of electronic patient records. British Journal of General Practice. 2018 Jun;68(671):e388–93.

24. Smith T, Fell C, Otete H, Chauhan U. GP incentives to design hypertension and atrial fibrillation local quality-improvement schemes: a controlled before–after study in UK primary care. Br J Gen Pract. 2019 Oct 1;69(687):e689–96.

25. Schwartz LM, Woloshin S, Floyd J. Fowler J, Welch HG. Enthusiasm for Cancer Screening in the United States. JAMA. 2004 Jan 7;291(1):71–8.

26. Waller J, Osborne K, Wardle J. Enthusiasm for cancer screening in Great Britain: a general population survey. Br J Cancer. 2015 Feb 3;112(3):562–6.

27. Lown M, Wilcox CR, Hughes S, Santer M, Lewith G, Moore M, et al. Patients' views about screening for atrial fibrillation (AF): a qualitative study in primary care. BMJ Open. 2020 Mar 1;10(3):e033061.

For peer review only

28. Bury G, Swan D, Cullen W, Keane D, Tobin H, Egan M, et al. Screening for atrial fibrillation in general practice: A national, cross-sectional study of an innovative technology. International Journal of Cardiology. 2015 Jan 15;178:247–52.

29. Malterud K, Siersma VD, Guassora AD. Sample Size in Qualitative Interview Studies: Guided by Information Power. Qualitative Health Research. 2016 Nov;26(13):1753–60.

30. Howson A. Cervical screening, compliance and moral obligation. Sociology of Health & Illness. 1999;21(4):401–25.

31. Bush J. "It's just part of being a woman": cervical screening, the body and femininity. Social Science & Medicine. 2000 Feb 1;50(3):429–44.

32. Ward PR, Coffey C, Meyer S. Trust, choice and obligation: a qualitative study of enablers of colorectal cancer screening in South Australia. Sociology of Health & Illness. 2015;37(7):988–1006.

33. Howson A. Embodied obligation: The female body and health surveillance. In: Nettleton S, Watson J, editors. The body in everyday life. ProQuest Ebook Central, http://ebookcentral.proquest.com: Taylor and Francis; 1998.

34. Willis K. Personal choice/social responsibility: Women aged 40–49 years and mammography screening. Journal of Sociology. 2004 Jun;40(2):121–36.

35. Willis K. "I Come Because I Am Called": Recruitment and Participation in Mammography Screening in Uppsala, Sweden. Health Care for Women International. 2008 Feb 11;29(2):135–50.

36. Armstrong N. Navigating the uncertainties of screening: the contribution of social theory. Soc Theory Health. 2019 Jun 1;17(2):158–71.

37. Griffiths F, Bendelow G, Green E, Palmer J. Screening for breast cancer: Medicalization, visualization and the embodied experience: Health: [Internet]. 2010 Oct 25 [cited 2020 Oct 14]; Available from: https://journals.sagepub.com/doi/10.1177/1363459310361599?url_ver=Z39.88-2003&rfr_id=ori%3Arid%3Acrossref.org&rfr_dat=cr_pub++0pubmed

38. Bikker AP, Macdonald S, Robb KA, Conway E, Browne S, Campbell C, et al. Perceived colorectal cancer candidacy and the role of candidacy in colorectal cancer screening. Health, Risk & Society. 2019 Nov 17;21(7–8):352–72.

39. Turk A, Boylan A-M, Locock L. A Researcher's Guide to Patient and Public Involvement [Internet]. University of Oxford; [cited 2021 Oct 1]. Available from: https://oxfordbrc.nihr.ac.uk/wp-content/uploads/2017/03/A-Researchers-Guide-to-PPI.pdf

40. Sheridan R, Martin-Kerry J, Hudson J, Parker A, Bower P, Knapp P. Why do patients take part in research? An overview of systematic reviews of psychosocial barriers and facilitators. Trials [Internet]. 2020 Mar 12 [cited 2020 Oct 21];21. Available from: https://www.ncbi.nlm.nih.gov/pmc/articles/PMC7069042/

41. Rebolj M, Parmar D, Maroni R, Blyuss O, Duffy SW. Concurrent participation in screening for cervical, breast, and bowel cancer in England. J Med Screen. 2020 Mar;27(1):9–17.

For peer review only

42. McCowan C, McSkimming P, Papworth R, Kotzur M, McConnachie A, Macdonald S, et al. Comparing uptake across breast, cervical and bowel screening at an individual level: a retrospective cohort study. British Journal of Cancer. 2019;121(8):710–4.

43. Public Health England. Supporting the health system to reduce inequalities in screening. https://assets.publishing.service.gov.uk/government/uploads/system/uploads/attachment_data/file/816245/PHE_Screening_inequalities_strategy_2018__1_.pdf: Public Health England; 2019 p. 27. Report No.: GW-489.

44. Lip GYH, Kamath S, Jafri M, Mohammed A, Bareford D. Ethnic Differences in Patient Perceptions of Atrial Fibrillation and Anticoagulation Therapy. Stroke. 2002 Jan 1;33(1):238–42.

45. McCabe PJ, Barton DL, DeVon HA. Older Adults at Risk for Atrial Fibrillation Lack Knowledge and Confidence to Seek Treatment for Signs and Symptoms. SAGE Open Nurs [Internet]. 2017 [cited 2020 Oct 6];3. Available from: https://www.ncbi.nlm.nih.gov/pmc/articles/PMC6326385/

46. McCabe PJ, Rhudy LM, DeVon HA. Patients' experiences from symptom onset to initial treatment for atrial fibrillation. Journal of Clinical Nursing. 2015;24(5–6):786–96.

47. Aliot E, Breithardt G, Brugada J, Camm J, Lip GYH, Vardas PE, et al. An international survey of physician and patient understanding, perception, and attitudes to atrial fibrillation and its contribution to cardiovascular disease morbidity and mortality. Europace. 2010 May;12(5):626–33.

48. Koponen L, Rekola L, Ruotsalainen T, Lehto M, Leino-Kilpi H, Voipio-Pulkki L-M. Patient knowledge of atrial fibrillation: 3-month follow-up after an emergency room visit. Journal of Advanced Nursing. 2008;61(1):51–61.

49. Mehawej J, Saczynski J, Gurwitz JH, Abu HO, Bamgbade BA, WANG W-J, et al. Self-reported risk of stroke and factors associated with underestimation of stroke risk among older adults with atrial fibrillation: the SAGE-AF study. J Geriatr Cardiol. 2020 Aug;17(8):502–9.

50. Hoffmann TC, Del Mar C. Patients' expectations of the benefits and harms of treatments, screening, and tests: a systematic review. JAMA Intern Med. 2015 Feb;175(2):274–86.

51. Gigerenzer G, Mata J, Frank R. Public knowledge of benefits of breast and prostate cancer screening in Europe. Journal of the National Cancer Institute. 2009 Sep 2;101(17):1216–20.

52. Kotwal AA, Walter LC, Lee SJ, Dale W. Are We Choosing Wisely? Older Adults' Cancer Screening Intentions and Recalled Discussions with Physicians About Stopping. Journal of General Internal Medicine. 2019;34(8):1538–45.

53. Torke AM, Schwartz PH, Holtz LR, Montz K, Sachs GA. Older adults and forgoing cancer screening: 'I think it would be strange'. JAMA internal medicine. 2013 Apr 8;173(7):526–31.

54. Richardson JC, Hassell AB, Hay EM, Thomas E. `I'd rather go and know': women's understanding and experience of DEXA scanning for osteoporosis. Health Expect. 2002 Jun;5(2):114–26.

55. Silverman E, Woloshin S, Schwartz LM, Byram SJ, Welch HG, Fischhoff B. Women's Views on Breast Cancer Risk and Screening Mammography: A Qualitative Interview Study. Med Decis Making. 2001 May;21(3):231–40.

For peer review only

56. Green E, Griffiths F, Thompson D. 'Are My Bones Normal Doctor?' the Role of Technology in Understanding and Communicating Health Risks for Midlife Women. Sociological Research Online. 2006 Dec;11(4):1–16.

57. Sutkowi-Hemstreet A, Vu M, Harris R, Brewer NT, Dolor RJ, Sheridan SL. Adult Patients' Perspectives on the Benefits and Harms of Overused Screening Tests: a Qualitative Study. Journal of General Internal Medicine. 2015 Nov;30(11):1618–26.

58. Stiggelbout A, Copp T, Jacklyn G, Jansen J, Liefers G-J, McCaffery K, et al. Women's Acceptance of Overdetection in Breast Cancer Screening: Can We Assess Harm-Benefit Tradeoffs? Medical Decision Making: An International Journal of the Society for Medical Decision Making. 2020;40(1):42–51.

59. Tacken MAJB, Braspenning JC, Hermens RP, Spreeuwenberg PM, van den Hoogen HJ, de Bakker DH, et al. Uptake of cervical cancer screening in The Netherlands is mainly influenced by women's beliefs about the screening and by the inviting organization [Internet]. Vol. 17, European journal of public health. Eur J Public Health; 2007 [cited 2020 Oct 14]. Available from: https://pubmed.ncbi.nlm.nih.gov/16837520/

60. Douma LN, Uiters E, Verweij MF, Timmermans DRM. Autonomous and informed decision-making: The case of colorectal cancer screening. PloS One. 2020;15(5):e0233308.

61. Hulme J, Moravac C, Ahmad F, Cleverly S, Lofters A, Ginsburg O, et al. "I want to save my life": Conceptions of cervical and breast cancer screening among urban immigrant women of South Asian and Chinese origin. BMC Public Health [Internet]. 2016 Oct 13 [cited 2020 Nov 5];16. Available from: https://www.ncbi.nlm.nih.gov/pmc/articles/PMC5062908/

62. Hope KA, Moss E, Redman CWE, Sherman SM. Psycho-social influences upon older women's decision to attend cervical screening: A review of current evidence. Prev Med. 2017 Aug;101:60–6.

63. Llanwarne N, Newbould J, Burt J, Campbell JL, Roland M. Wasting the doctor's time? A video-elicitation interview study with patients in primary care. Social Science & Medicine. 2017 Mar 1;176:113–22.

64. Angela E Raffle, Mackie A, Gray JAM. Screening: Evidence and Practice. Second edition. Oxford: Oxford University Press;

65. Gillespie C. The experience of risk as 'measured vulnerability': health screening and lay uses of numerical risk. Sociology of Health & Illness. 2012;34(2):194–207.

66. Deaton C, Dunbar SB, Moloney M, Sears SF, Ujhelyi MR. Patient experiences with atrial fibrillation and treatment with implantable atrial defibrillation therapy. Heart & Lung. 2003 Sep 1;32(5):291–9.

67. Zweiker D, Zweiker R, Winkler E, Roesch K, Schumacher M, Stepan V, et al. Association between subjective risk perception and objective risk estimation in patients with atrial fibrillation: a cross-sectional study. BMJ Open [Internet]. 2017 Sep 25 [cited 2020 Oct 6];7(9). Available from: https://www.ncbi.nlm.nih.gov/pmc/articles/PMC5623506/

68. Kaufman BG, Kim S, Pieper K, Allen LA, Gersh BJ, Naccarelli GV, et al. Disease understanding in patients newly diagnosed with atrial fibrillation. Heart. 2018 Mar;104(6):494–501.

For peer review only

69. Barnett KN, Weller D, Smith S, Steele RJ, Vedsted P, Orbell S, et al. The contribution of a negative colorectal screening test result to symptom appraisal and help-seeking behaviour among patients subsequently diagnosed with an interval colorectal cancer. Health Expectations. 2018;21(4):764–73.

70. Deutekom M, Vansenne F, McCaffery K, Essink-Bot M-L, Stronks K, Bossuyt PMM. The effects of screening on health behaviour: a summary of the results of randomized controlled trials. J Public Health (Oxf). 2011 Mar 1;33(1):71–9.

71. Tymstra T, Bieleman B. The Psychosocial Impact of Mass Screening for Cardiovascular Risk Factors. Fam Pract. 1987 Dec 1;4(4):287–90.

72. Solbjør M, Skolbekken J-A, Sætnan AR, Hagen AI, Forsmo S. Could screening participation bias symptom interpretation? An interview study on women's interpretations of and responses to cancer symptoms between mammography screening rounds. BMJ Open. 2012 Jan 1;2(6):e001508.

73. Public Health England. Public Health Profiles [Internet]. [cited 2021 Oct 5]. Available from: https://fingertips.phe.org.uk/

74. National Statistics. English indices of deprivation 2019 [Internet]. GOV.UK. [cited 2021 Oct 5]. Available from: https://www.gov.uk/government/statistics/english-indices-of-deprivation-2019

For peer review only

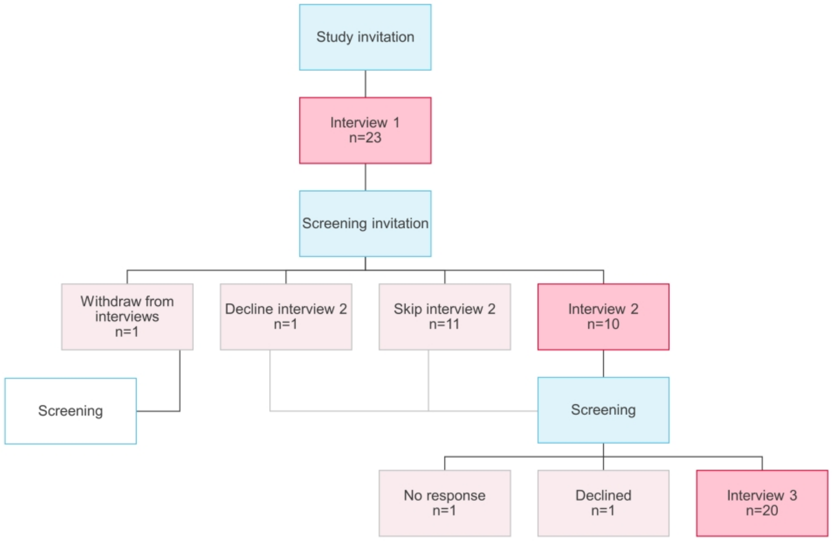

Figure 1: Flowchart of interviews in the screening process

338x190mm (300 x 300 DPI)

SAFER study patient interview topic guide

Introduction and Consent Process

- Researcher to introduce themselves and check understanding of study and processes
- Informed consent and permission to audio record the interview obtained from the participant and partner (or other) if present
- (If applicable) - Explain to patient that this interview is like the previous interview they completed but will now focus on either their views about the screening invitation or their experience of screening.
- Select schedule based on time point of interview.

Interview 1 (post SAFER study invitation)

A. Experiences of screening

I'd like to start by thinking quite generally about screening programmes. There are national screening programmes for many different conditions – things like breast cancer, bowel cancer, and abdominal aortic aneurysm screening. What do you think about screening programmes?

Prompts:

- *Why do you think screening programmes are offered by the NHS?*
- *Potential benefits of screening*
- *Potential harms of screening*

Have you had experience of one of these screening programmes? I'm not asking for you to reveal personal information, just wanting to see if you had decided to go along to screening for something in response to an invitation.

Prompts:

- *Positive or negative experiences [if seemingly acceptable]*

B. Focus on atrial fibrillation

OK, we are going to move on to thinking about this particular condition – atrial fibrillation.

Had you ever heard of atrial fibrillation before you received the study invite? I'm not expecting you to know anything about it, just wanted to check in in case you do.

Prompts:

- *Does anyone they know have AF?*
- *Have they heard anything about AF from any other source?*

[Researcher to explain about atrial fibrillation]:

"Atrial fibrillation (AF) is a condition where the heart has an irregular beat. It may not cause symptoms, occurs in up to 10% of people over 65 years old. AF greatly increases risk of stroke by increasing the chance of blood clots developing. However, there is treatment that inhibits blood clotting (an anticoagulant, sometimes known as 'blood thinners'), and reduces the risk of stroke. About 10% of strokes occur in people unaware that they have AF."

For peer review only

What do you think having AF might mean for someone?
Prompts:
- *What it might be like to have an irregular heart beat*
- *Fears or concerns about living with AF*

What would you think about taking regular 'blood thinning' medication for AF?
Prompts:
- *An example medication is warfarin – what do you think about this medication?*

What do you think about the link AF has with stroke?
Prompts:
- *What experiences do people have with stroke?*
- *What are people's reactions to or concerns about stroke?*
- *What might it be like living with AF, knowing it increases the risk of stroke?*

C. Any other areas of discussion

Do you have any other areas, which we have not yet covered, which you would like to discuss in relation to this topic?

Interview close

- Thank participants for taking part in the study and remind them that their responses will remain confidential
- Ask participants if they have any questions.
- Let participants know they can contact the research team at any time using the details on the information leaflet, including if /d they have any further questions or wish to remove their responses from the study.

For peer review only

Interview 2 (post screening invitation)

A. Screening for atrial fibrillation

As you know, this interview is part of a research study we are undertaking to explore the offer of screening for AF in primary care. What were your first impressions on receiving the invitation to be screened for AF?

Prompts

- *Did you know anything about AF before you received the screening invite?*
- *Did you feel there was sufficient information in the screening invite?*

Do you think there might be any benefits of screening for AF?

Prompts:

- *Potential treatment of AF*
- *Stroke prevention*

Do you think there might be any harms or negative consequences of screening for AF?

Prompts:

- *Potential harms: psychological, false-positives, overdiagnosis, overtreatment, reduced quality of life, financial, opportunity costs*

Do you think this screening is relevant to you?

Prompt:

- *If so, why?*
- *If not, why not?*

Are you planning to go along for screening?

Prompt:

- *If so, why?*
- *If not, why not?*

Would you change anything about your life if screening showed you did have AF?

Prompt:

- *Lifestyle changes (routine/exercise/diet/smoking)*

B. Any other areas of discussion

Do you have any other areas, which we have not yet covered, which you would like to discuss in relation to this topic?

For peer review only

Interview 3 (Post screening)

A. Experiences of screening for AF
I'd like to explore with you your experiences of the AF screening programme. What were your first impressions on receiving the invitation to be screened for AF?
Prompts
- *Did you know anything about AF before you received the screening invite?*
- *Did you feel there was sufficient information in the screening invite?*
- *Did you feel the screening was relevant to you?*
- *If declined, why was this? What were your concerns about the process?*

Now I'd like to talk a little bit about what happened during the screening process [to be asked of screening attenders]
Prompts
- *What prompted you to go along?*
- *Did you understand what screening might involve?*
- *Did the doctor/nurse explain it all clearly to you, do you think?*
- *How did you find using the Zenicor device?*
- *Did you have any particular difficulties or challenges taking the readings you were asked to complete?*
- *Were you concerned about anything during the screening process? What was this in particular? Did you let anyone know about your concerns? Were these concerns addressed?*
- *What was good about your overall experience? Was there anything that was not so good about your experience?*
- *Did you understand how you were going to be informed about the outcome of your screening? If not, why not? How could things have been done differently?*
- *How did taking part in the screening make you feel?*

Given your experiences, do you think there are any benefits of screening for AF?
Prompts:
- *Potential treatment of AF*
- *Stroke prevention*

Do you think there might be any harms or negative consequences of screening for AF?
Prompts:
- *Potential harms: psychological, false-positives, overdiagnosis, overtreatment, reduced quality of life, financial, opportunity costs*

Would you recommend this screening programme to a find or family member?
Prompts:
- *If so, why?*
- *If not, why not?*

C. Any other areas of discussion

Do you have any other areas, which we have not yet covered, which you would like to discuss in relation to this topic?

For peer review only

COREQ (COnsolidated criteria for REporting Qualitative research) Checklist

A checklist of items that should be included in reports of qualitative research. You must report the page number in your manuscript where you consider each of the items listed in this checklist. If you have not included this information, either revise your manuscript accordingly before submitting or note N/A.

Topic	Item No.	Guide Questions/Description	Reported on Page No.
**Domain 1: Research team and reflexivity**			
Personal characteristics			
Interviewer/facilitator	1	Which author/s conducted the interview or focus group?	
Credentials	2	What were the researcher's credentials? E.g. PhD, MD	
Occupation	3	What was their occupation at the time of the study?	
Gender	4	Was the researcher male or female?	
Experience and training	5	What experience or training did the researcher have?	
Relationship with participants			
Relationship established	6	Was a relationship established prior to study commencement?	
Participant knowledge of the interviewer	7	What did the participants know about the researcher? e.g. personal goals, reasons for doing the research	
Interviewer characteristics	8	What characteristics were reported about the inter viewer/facilitator? e.g. Bias, assumptions, reasons and interests in the research topic	
**Domain 2: Study design**			
Theoretical framework			
Methodological orientation and Theory	9	What methodological orientation was stated to underpin the study? e.g. grounded theory, discourse analysis, ethnography, phenomenology, content analysis	
Participant selection			
Sampling	10	How were participants selected? e.g. purposive, convenience, consecutive, snowball	
Method of approach	11	How were participants approached? e.g. face-to-face, telephone, mail, email	
Sample size	12	How many participants were in the study?	
Non-participation	13	How many people refused to participate or dropped out? Reasons?	
Setting			
Setting of data collection	14	Where was the data collected? e.g. home, clinic, workplace	
Presence of non-participants	15	Was anyone else present besides the participants and researchers?	
Description of sample	16	What are the important characteristics of the sample? e.g. demographic data, date	
Data collection			
Interview guide	17	Were questions, prompts, guides provided by the authors? Was it pilot tested?	
Repeat interviews	18	Were repeat inter views carried out? If yes, how many?	
Audio/visual recording	19	Did the research use audio or visual recording to collect the data?	
Field notes	20	Were field notes made during and/or after the inter view or focus group?	
Duration	21	What was the duration of the inter views or focus group?	
Data saturation	22	Was data saturation discussed?	
Transcripts returned	23	Were transcripts returned to participants for comment and/or	

For peer review only

Topic	Item No.	Guide Questions/Description	Reported on Page No.
		correction?	
**Domain 3: analysis and findings**			
Data analysis			
Number of data coders	24	How many data coders coded the data?	
Description of the coding tree	25	Did authors provide a description of the coding tree?	
Derivation of themes	26	Were themes identified in advance or derived from the data?	
Software	27	What software, if applicable, was used to manage the data?	
Participant checking	28	Did participants provide feedback on the findings?	
Reporting			
Quotations presented	29	Were participant quotations presented to illustrate the themes/findings? Was each quotation identified? e.g. participant number	
Data and findings consistent	30	Was there consistency between the data presented and the findings?	
Clarity of major themes	31	Were major themes clearly presented in the findings?	
Clarity of minor themes	32	Is there a description of diverse cases or discussion of minor themes?	

Developed from: Tong A, Sainsbury P, Craig J. Consolidated criteria for reporting qualitative research (COREQ): a 32-item checklist for interviews and focus groups. *International Journal for Quality in Health Care*. 2007. Volume 19, Number 6: pp. 349 – 357

Once you have completed this checklist, please save a copy and upload it as part of your submission. DO NOT include this checklist as part of the main manuscript document. It must be uploaded as a separate file.

For peer review only
